# Identification of superior and rare haplotypes to optimize branch number in soybean

**DOI:** 10.1007/s00122-024-04596-y

**Published:** 2024-04-03

**Authors:** Hui Yu, Javaid Akhter Bhat, Candong Li, Beifang Zhao, Moran Bu, Zhirui Zhang, Tai Guo, Xianzhong Feng

**Affiliations:** 1 Key Laboratory of Soybean Molecular Design Breeding, State Key Laboratory of Black Soils Conservation and Utilization, Northeast Institute of Geography and Agroecology, Chinese Academy of Sciences, Changchun, 130102 China; 2 Zhejiang Lab, Hangzhou, 310012 China; 3 Jiamusi Branch Academy of Heilongjiang Academy of Agricultural Sciences, Jiamusi, 154007 China; 4 College of Advanced Agricultural Sciences, University of Chinese Academy of Sciences, Beijing, 101408 China

## Abstract

**Key message:**

**Using the integrated approach in the present study, we identified eleven significant SNPs, seven stable QTLs and 20 candidate genes associated with branch number in soybean**.

**Abstract:**

Branch number is a key yield-related quantitative trait that directly affects the number of pods and seeds per soybean plant. In this study, an integrated approach with a genome-wide association study (GWAS) and haplotype and candidate gene analyses was used to determine the detailed genetic basis of branch number across a diverse set of soybean accessions. The GWAS revealed a total of eleven SNPs significantly associated with branch number across three environments using the five GWAS models. Based on the consistency of the SNP detection in multiple GWAS models and environments, seven genomic regions within the physical distance of ± 202.4 kb were delineated as stable QTLs. Of these QTLs, six QTLs were novel, viz., *qBN7*, *qBN13, qBN16, qBN18, qBN19 and qBN20*, whereas the remaining one, viz., *qBN12*, has been previously reported. Moreover, 11 haplotype blocks, viz., Hap4, Hap7, Hap12, Hap13A, Hap13B, Hap16, Hap17, Hap18, Hap19A, Hap19B and Hap20, were identified on nine different chromosomes. Haplotype allele number across the identified haplotype blocks varies from two to five, and different branch number phenotype is regulated by these alleles ranging from the lowest to highest through intermediate branching. Furthermore, 20 genes were identified underlying the genomic region of ± 202.4 kb of the identified SNPs as putative candidates; and six of them showed significant differential expression patterns among the soybean cultivars possessing contrasting branch number, which might be the potential candidates regulating branch number in soybean. The findings of this study can assist the soybean breeding programs for developing cultivars with desirable branch numbers.

**Supplementary Information:**

The online version contains supplementary material available at 10.1007/s00122-024-04596-y.

## Introduction

Soybean [*Glycine max* (L.) Merr.] is a popular legume crop grown globally as it is a rich source of edible oil and protein (Zhang et al. 2019a, b). In addition, this crop has roles in soil fertility, biofuel and human health (Thapa et al. 2021). China is the largest consumer of soybean and its commercial products are increasingly dependent on soybean imports (Yu et al. 2023). In the past five decades, soybean yield improvement efforts have been almost stagnant in China (Bhat et al. 2022a). Hence, there is a great need for China to increase domestic production to make the country self-sufficient in soybean production. Breeders target different yield-related traits to increase soybean production. In this regard, branch number per plant is an important trait related to the plant architecture, adaptability and yield of soybean (Shim et al. 2017, 2019). Studies have documented the significant correlation between branch number and yield in soybean (Lu et al. 2017). Hence, it is the core objective of soybean breeders to develop cultivars with desirable branch numbers. Moreover, depending on the environmental and cost conditions, soybean cultivars with different branching patterns can be selected for planting (Shim et al. 2017). For example, under conditions of disease and stalk lodging issues, a lower plant density is preferred, which in turn decreases labor and seed costs (Cho and Kim 2010). In addition, cultivars with higher branch numbers can be used for planting in these conditions because they can compensate for the lower sowing rate (Cox et al. 2010). In contrast, in the case of dense planting, cultivars with fewer branches are preferred (Agudamu and Shiraiwa 2016). Based on the phenotypic data from the Germplasm Resources Information Network (GRIN, http://www.ars-grin.gov/), considerable differences in branch number have been observed among soybean varieties (Sayama et al. 2010). This has revealed that substantial genetic diversity in terms of branch number exists among soybean accessions and can be utilized in soybean improvement. Regulation of branching at the genetic level has also remained an interesting topic in plant developmental biology in addition to its importance in soybean breeding for enhancing yield (Sayama et al. 2010). Therefore, it is necessary to elucidate the detailed genetic basis of branch number for the production of soybean cultivars with desirable branch numbers. Knowledge about the genes controlling branch number in soybean will allow their deployment in breeding for producing soybean cultivars with desirable branch numbers (Zhang et al. 2019a, b).

Despite the great pace of genomic research in soybean over the last few decades, the branch number trait has been almost neglected, and only ~ 22 genetic loci have been documented for branch number in SoyBase (https://www.soybase.org/). Among these QTLs, no single one has been confirmed and deployed in marker-assisted breeding. This disadvantage is due to the use of conventional linkage mapping approaches and low-density markers for the QTL identification of branch number in previous studies. However, the high resolution of GWAS together with advances in sequencing and statistical models has made marker-assisted breeding practical in crop improvement (Schmutz et al. 2010; Bhat and Yu 2021). In recent years, advances in sequencing and high-throughput genotyping have allowed the routine use of GWAS in crop plants. Multiple studies have demonstrated the performance of GWAS in gene mapping in wheat (Saini et al. 2022), soybean (Bhat et al. 2022b; Yu et al. 2023), maize (Ma et al. 2022), *Arabidopsis thaliana* (Sasaki et al. 2022), chickpea (Thudi et al. 2021) and rice (Lv et al. 2022).

The GWAS approach has been thoroughly validated for its potential in unraveling the genetic architecture of complex quantitative traits in crop plants (Alqudah et al. 2020). However, until now, GWAS has mostly entailed the use of biallelic SNP markers, which have the disadvantage of not capturing rare/superior alleles as well as epistatic variation (Yu et al. 2023). Rare alleles present in crop germplasm are often responsible for the superior phenotype for the trait of interest (Bhat et al. 2021). In this context, the analysis of multiallelic markers such as haplotypes can capture this variation and make it utilizable for crop improvement (Bhat et al. 2021). Both simulation and empirical studies have shown better performance of haplotype markers relative to SNP markers (Luján Basile et al. 2019; Yu et al. 2023). Multiple features of haplotype markers, including their multiallelic nature, their potential of capturing epistatic variation and rare alleles, their higher number of haplotype variants, their controlling of false positives/negatives and their higher polymorphism information content (PIC) value, can contribute to their superior performance in GWAS (Bhat et al. 2021). In comparison with SNP markers, haplotypes have been documented to increase the phenotypic variation explained (PVE) by 50% and the allelic effect by 34% (Hamblin and Jannink 2011). It has also been reported that relative to SNP markers, which have shown a PIC of 0.27, haplotype markers showed an increased PIC of 0.50 in wheat crops (N’Diaye et al. 2017). Abbai et al. (2019) identified superior haplotypes for yield-related and quality traits in rice by using the GWAS approach in a 3000 rice accession panel. In other crops, such as pigeonpea, soybean and rice, superior haplotypes have been identified for stress tolerance (Guan et al. 2014; Kuroha et al. 2018; Sinha et al. 2020; Yu et al. 2023).

To elucidate the genetic makeup of the branch number in soybean, we conducted an integrated approach with GWAS, haplotype analysis and candidate gene identification in a diverse set of soybean accessions collected from five accumulated temperature zones of northeastern China. Our study presents the identified superior haplotypes, stable QTLs and candidate genes regulating branch number in soybean. The results of our study can be successfully deployed in marker-assisted breeding of desired branch number in soybean, although some initial validation will be needed for the functional verification of candidate genes.

## Materials and methods

### Plant materials and experimental conditions

In the current study, we used a diverse collection of 200 soybean cultivars collected from northeastern China, and this collection of accessions represents five temperature accumulated zones. In each accumulated temperature zone, the temperature remains constant (Table S1). Two locations in different provinces, viz., Jiamusi and Jilin, were used for phenotypic evaluation of the soybean germplasm. Jiamusi is in Heilongjiang Province, China, and in this location, the germplasm collection was evaluated in two consecutive years, viz., 2017 and 2018 representing two environments, JMS17 (Jiamusi 2017) and JMS18 (Jiamusi 2018), respectively. Jiamusi (46.82° N, 130.37° E) has very warm summers, and the winters are long, dry and bitter. Jiamusi receives an average rainfall of 725.3 mm, with an average temperature of 15.4 °C and an average relative humidity of 62.8%. The Jilin location is in central Jilin Province and spans the area from 125° 40' to 127° 56' E longitude and 42° 31' to 44° 40' N latitude. Jilin has four seasons: winters are long (November to March), cold and dry; spring and autumn are somewhat short transitional periods, with some precipitation, but are usually dry and windy; and summers are hot and humid. The average rainfall, average humidity and average temperature in Jilin are 370–410 mm, 65.81% and 16 ~ 24 °C, respectively. In the Jilin location, the germplasm collection was evaluated in 2020, representing the third environment JL20 (Jilin 2020). The planting of the germplasm collection was performed according to the randomized complete block design (RCBD), with the germplasm planted in three replicates. Each genotype was planted in three rows with a row length of 200 cm, and the spacing between rows was 50 cm. The seeds sown in each row consist of ~ 20–25, and more than 95% of the seeds germinated and reached to the maturity. Standard agronomic practices were followed to grow the soybean plants.

### Phenotypic evaluation and statistical analysis

Phenotypic data for branch number were collected by randomly selecting five plants from the middle row for each genotype at the maturity stage. Phenotypic collection for branch number was performed manually. The average branch number was used for the final analysis, and it was calculated by first taking the average for five plants selected from each replicate and then taking the average for the three replicates. The data obtained from individual environments were used to estimate the combined environment data using “*lme4*” package in R environment following the Bhat et al. (2022a). The predicted means (BLUPs) for the combined environment were estimated using the following model:

$$Y_{ijk} = \mu + {\text{ Env}}_{i} + {\text{ Rep}}_{j} \left( {{\text{ Env}}_{i} } \right) \, + {\text{ Gen}}_{k} + {\text{ Env}}_{i} \times {\text{ Gen}}_{k} + \varepsilon_{ijk}$$

where *Y*_*ijk*_ represents the branch number per plant, *µ* represents overall mean effect, and Rep_*i*_ is the effect of the *i*th replicate/block. Gen_*j*_ is effect of the *j*th genotype; ε_*ij*_ represents effect of the error associated with the *i*th replication/block, and *j*th genotype; where Env_*i*_ and Env_*i*_ × Gen_*k*_ are the *i*th environment and the *G* × *E* interaction effects, respectively.

Descriptive statistical analysis involving the parameters of mean, range, kurtosis, skewness, standard deviation (SD) and coefficient of variation (CV%) for branch number was estimated using R software, and the specific function with proper summary statistics was used. In R software, the *aov* function was used to carry out the analysis of variance (ANOVA). Broad-sense heritability (*h*^*2*^) was calculated by using the equation below:

$$h^{2} = \sigma^{2}_{g} /\left( {\sigma^{2}_{g} + \sigma^{2}_{ge} /n + \sigma^{2}_{e} /{\text{nr}}} \right)$$

where *σ*^*2*^_*g*,_ *σ*^*2*^_*ge*,_ *σ*^*2*^_*e*_, *n* and* r* represent the genotypic variance, genotype-by-environment interaction variance, error variance, number of environments and number of replicates, respectively (Nyquist and Baker 1991).

### SNP selection and genotyping

Fresh and healthy soybean leaf tissues from three-week-old soybean seedlings were used to extract DNA. Murray and Thompson’s (1980) cetyltrimethylammonium bromide (CTAB) method was used for DNA extraction. An insert size of ~ 350 bp was used for the preparation of the library for each soybean accession, and the manufacturer’s instructions (Illumina Inc., San Diego, CA, USA) were followed to prepare the library. The Illumina HiSeq platform was used for the resequencing of the 200 soybean cultivars. High-quality SNPs were selected by following stringent quality control measures, such as a minor allele frequency (MAF) of 0.05 and missing genotype at 0.10 in VCFtools software v0.1.13 (Danecek et al. 2011). A total of 2,715,610 high-quality SNPs were ultimately identified that were used in subsequent experiments.

### Linkage disequilibrium and genome-wide association study

PopLDdecay software (Zhang et al. 2019a, b) was used to estimate genome-wide LD and to calculate the *r*^*2*^ (squared allele-frequency correlation) among the SNPs with known genomic positions. The expected values of *r*^*2*^ under drift equilibrium were also estimated by PopLDdecay software, and these expected values were plotted against the physical distance (kb) using the same software. Smoothing spline regression lines were used to fit the LD decay curve on a scatterplot at the genome level (Remington et al. 2001).

GWAS was performed using the Genomic Association and Prediction Integrated Tool version 3 (GAPIT3) (Lipka et al. 2012; Wang and Zhang 2021), where the SUPER (Wang et al. 2014), multiple locus MLM (MLMM), fixed and random model circulating probability unification (FarmCPU) (Liu et al. 2016), and Bayesian information and linkage disequilibrium iteratively nested keyway (BLINK) (Huang et al. 2019) were run in this study. All these four models, viz., MLMM, FarmCPU, SUPER and BLINK, are multi-locus models. All the models were executed by using the GAPIT v3 package (Lipka et al. 2012) in an R environment. To estimate the optimal number of PCA for correcting the population structure, PCA was employed using PLINK (Purcell et al. 2007). Based on the accumulated temperature zones, the population structure was visualized using the “ggplot2” package in the R environment. In GAPIT v3, the default method with the negative logarithm of the *P* value (where *P* value = 0.05/number of markers, and 0.05 represents MAF cutoff) was used to determine the significant SNP associations.

For using the 3VmrMLM method in the detection of marker-trait association and quantitative trait nucleotide (QTN) × environment interactions, we downloaded the R software IIIVmrMLM from GitHub website (https://github.com/YuanmingZhang65/IIIVmrMLM). In the current study, the multiple-environment method was used to detect QTNs and QTN × environment interactions. The significant threshold value was determined by LOD score ≥ 3.0.

### Haplotype analysis

The LD level among the SNP pairs was estimated using Haploview 4.2 (Barrett et al. 2005). The closest SNPs within the physical distance of ± 0 kb of the significant SNP represent the haplotype blocks. Haplotype blocks were defined by the “confidence intervals” algorithm (Gabriel et al. 2002). Soybean genotypes of the GWAS panel were grouped into separate groups based on the specific haplotype allele possessed by each genotype. To estimate the haplotype effect on branch number per plant, a one-way ANOVA model was used to fit the groups as follows:

$${\text{model}} \leftarrow {\text{aov}}({\text{phenotype}} \sim {\text{group}},{\text{ data}} = {\text{data}})$$

The phenotype is represented by the number of branches per plant in the combined environment. To compare the pairwise means, Tukey’s HSD test was used and visualized in the R environment.

### Candidate gene identification and qRT-PCR analysis

For candidate gene identification, we downloaded all the model genes within the physical interval of ± 202.4 kb of the significant SNP from SoyBase (https://www.soybase.org/) using the Williams 82 (*Wm82.a2.v1*) gene model. The annotations of these genes were also downloaded from SoyBase (https://www.soybase.org/). Based on the gene function annotation, variant annotation (synonymous/nonsynonymous) and literature search, the candidate genes underlying the physical interval of ± 202.4 kb of the significant SNP were selected (Yu et al. 2023).

The quantitative real-time PCR (qRT-PCR) was performed to analysis the gene expression patterns of candidate genes selected from above steps by using the five soybean cultivars with low branch number and four soybean cultivars with high branch number. The list of the soybean cultivars used for qRT-PCR analysis are presented in Table S2. The healthy and fresh leaf samples of soybean seedlings at V2 (second trifoliolate) stage were taken and ground in liquid nitrogen carefully by using a mortar and pestle. Total RNA was extracted using the TRIzol reagent mixture (Tiangen, Lot 118,721; China) by following the manufacturer’s instructions. The complementary DNA (cDNA) was synthesized using 4 μg of RNA as well as oligo (dT)18 primers and Moloney murine leukemia virus (M-MLV) reverse transcriptase (TransGen Lot N31204; China) according to the manufacturer’s protocol. Relative transcript levels were analyzed through real-time quantitative PCR (qRT–PCR) on an Mx3005P instrument (Stratagene, La Jolla, CA, United States) in conjunction with SYBR Green Master Mix (Genstar Lot 9BC01; China). The PCR parameters were 95 °C for 30 s (1 cycle), 95 °C for 5 s and 60 °C for 20 s (40 cycles), which was followed by a melting curve analysis at 95 °C for 60 s, 55 °C for 30 s and 95 °C for 30 s. The internal control gene *GmActin11* (*Glyma.18G290800*) was used for normalization of the transcript levels of genes in the samples (Yu et al. 2022). The relative fold differences were calculated via the 2^−ΔΔCt^ method. Three independent biological replicates were used to confirm the expression profiles. For genes expression comparison of different soybean cultivars, the FNG0853 genotype was set as the control. The specific primer pairs used are listed in Supplementary Table S3.

## Results

### Phenotypic analysis of branch number

The range (maximum and minimum value), mean, SD, CV kurtosis, skewness and *h*^*2*^ of the branch number in the GWAS panel of 200 accessions in the individual and combined environments are presented in Table S4. In the individual environments, the minimum and maximum values for branch number varied from 0.00 to 3.60. The mean value of branch number among the individual environments ranged from 0.50 ± 0.04 to 0.96 ± 0.05 in JMS17 and JMS18, respectively. The CV in the individual environments ranged from 68.44 to 121.92% in JMS18 and JMS17, respectively, and in the combined environment, the CV was 92.75%. In the combined environment, the kurtosis and skewness were 1.50 and 1.28, respectively (Table S4). A higher broad-sense heritability (*h*^*2*^) of 0.61 was observed in the combined environment. In addition, the genotype (*G*), environment (*E*) and genotype × environment interaction (*G* × *E*) variances of the branch number trait were highly significant (*P* < *0.0001*) in the GWAS panel (Table 1).

**Table 1:** Analysis of variance (ANOVA) for branch number in the combined environments

Source	DF	SS	MS	*F-value*	*P value* (Prob > *F*)
Genotype (*G*)	199	1025.9	5.16	12.016	< 2e^−16^
Environment (*E*)	2	113.8	56.88	132.595	< 2e^−16^
*G* × *E*	398	368.5	0.93	2.158	< 2e^−16^
Error	2400	1029.6	0.43		

*DF* degrees of freedom, *SS* sum of squares, *MS* mean sum of squares, *E* environment, *Prob* probability

*P* value < *0.0001,* significant

### Population structure and LD analysis

**Fig. 1:**
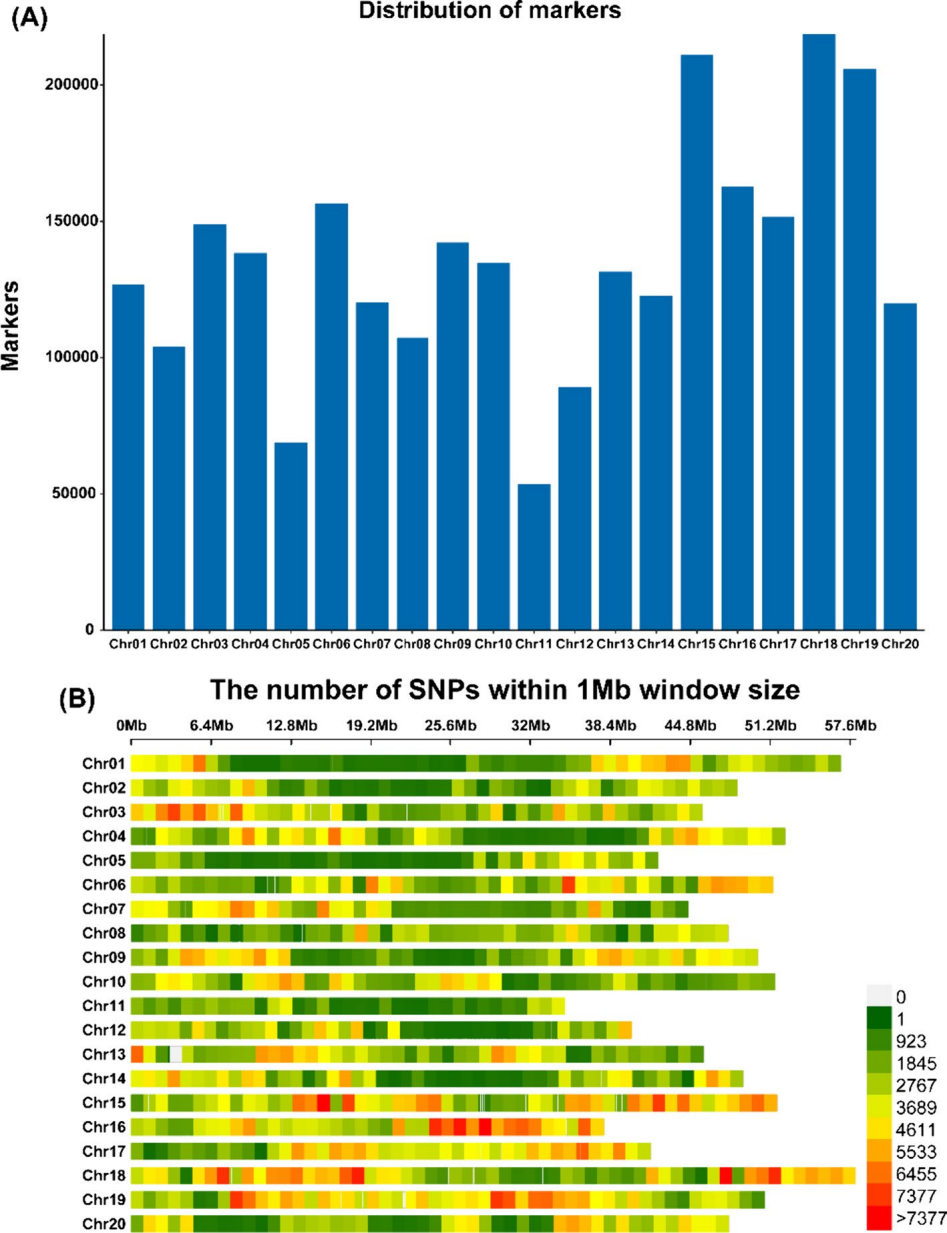
Marker distribution and density of 200 soybean accessions collected from five accumulated temperature zones of northeastern China. **A** Genome-wide distribution of 2,715,610 SNP markers that are used for GWAS. **B** This diagram shows the presence of the 2,715,610 SNPs across twenty soybean chromosomes. Length of chromosomes (Mb) is represented by the horizontal axis, chromosome number is denoted by the vertical axis, and SNP density is depicted by the different colours (number of SNPs per window)

**Fig. 2:**
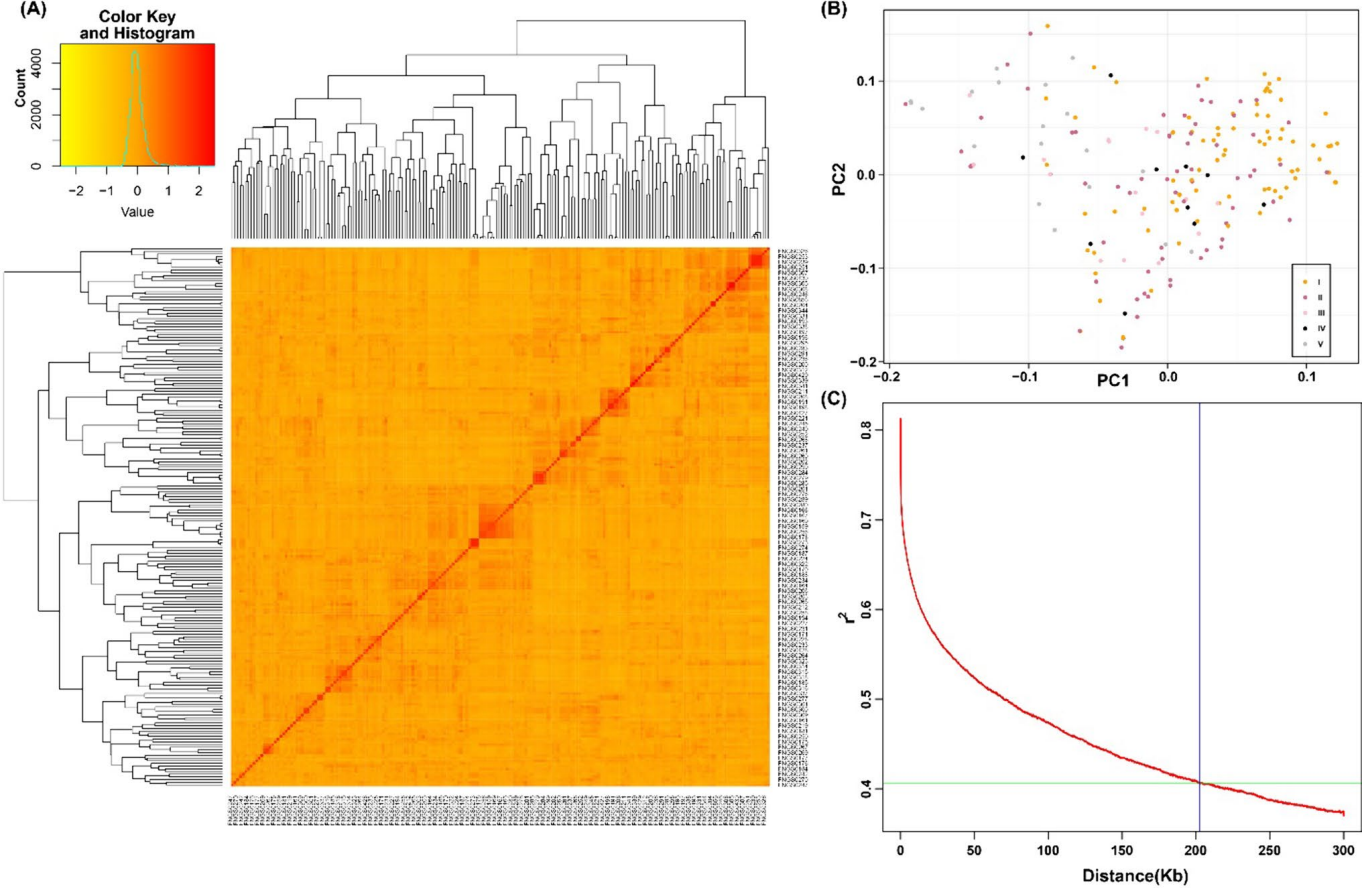
Kinship plot, population structure and whole-genome LD decay plot of 200 soybean accessions collected from five accumulated temperature zones of northeastern China. **A** Relationship of 200 soybean accessions depicted by a kinship plot. **B** Analysis of population structure for 200 soybean accessions collected from five accumulated temperature zones, and in each zone the temperature remained constant. **C** LD decay plot of 200 soybean cultivars using 2,715,610 SNP markers. The LD decay fitted with a smoothing spline regression model is represented by the red curve line. The blue vertical line intersection with the horizontal green line represents the half decay of LD (*r*^2^ = 0.405), and the genetic distance at this point corresponds to LD decay distance (202.4 kb)

The Illumina HiSeq platform was used for resequencing the 200 soybean accessions in the present study. Resequencing produced 150 bp paired-end reads that together comprises 3.3 trillion bases, with an average coverage depth of 16x. Reads of low quality, as well as reads with adaptors and “N”s, were removed, and only clean reads were retained. Mapping of the resequencing data with the reference genome of Williams 82 (*Wm82.a2.v1*) enabled the identification of a total of 4,523,188 SNPs and 673,692 Indels. By applying the quality control measures of MAF at 0.05 and missing genotype at 0.10, the final high-quality 2,715,610 SNPs were retained for further investigation. All 20 chromosomes were represented by these SNPs, and the highest and lowest SNP numbers were present on Chr.18 (218,699) and Chr.11 (53,502), respectively. Chr.11 and Chr.16 had the lowest and highest SNP densities of 1538.74 SNPs/Mb and 4299.02 SNPs/Mb, respectively (Fig. 1; Table S5). The heatmap and dendrogram of the kinship matrix were developed by using the above set of polymorphic SNPs, which revealed no clear clustering of the soybean accessions (Fig. 2A). In addition, a continuous distribution with no distinct structure was also revealed by population structure analysis (Fig. 2B; Table S1).

The GWAS panel of soybean accessions was also used to study the LD characteristics, and the results are presented graphically in Fig. 2C. Across the genome, the average *r*^*2*^ value was 0.46, and LD decay started at 0.81 and reached a half decay at 0.405. The intersection of the half decay with the LD decay curve occurred at 202.4 kb, and it is an important distance used to identify the linkage at the genome-wide level. The physical genomic interval within ± 202.4 kb represents the QTL region for significant SNPs detected by multiple models and environments.

### Marker–trait association (MTA) analysis for branch number

**Fig. 3:**
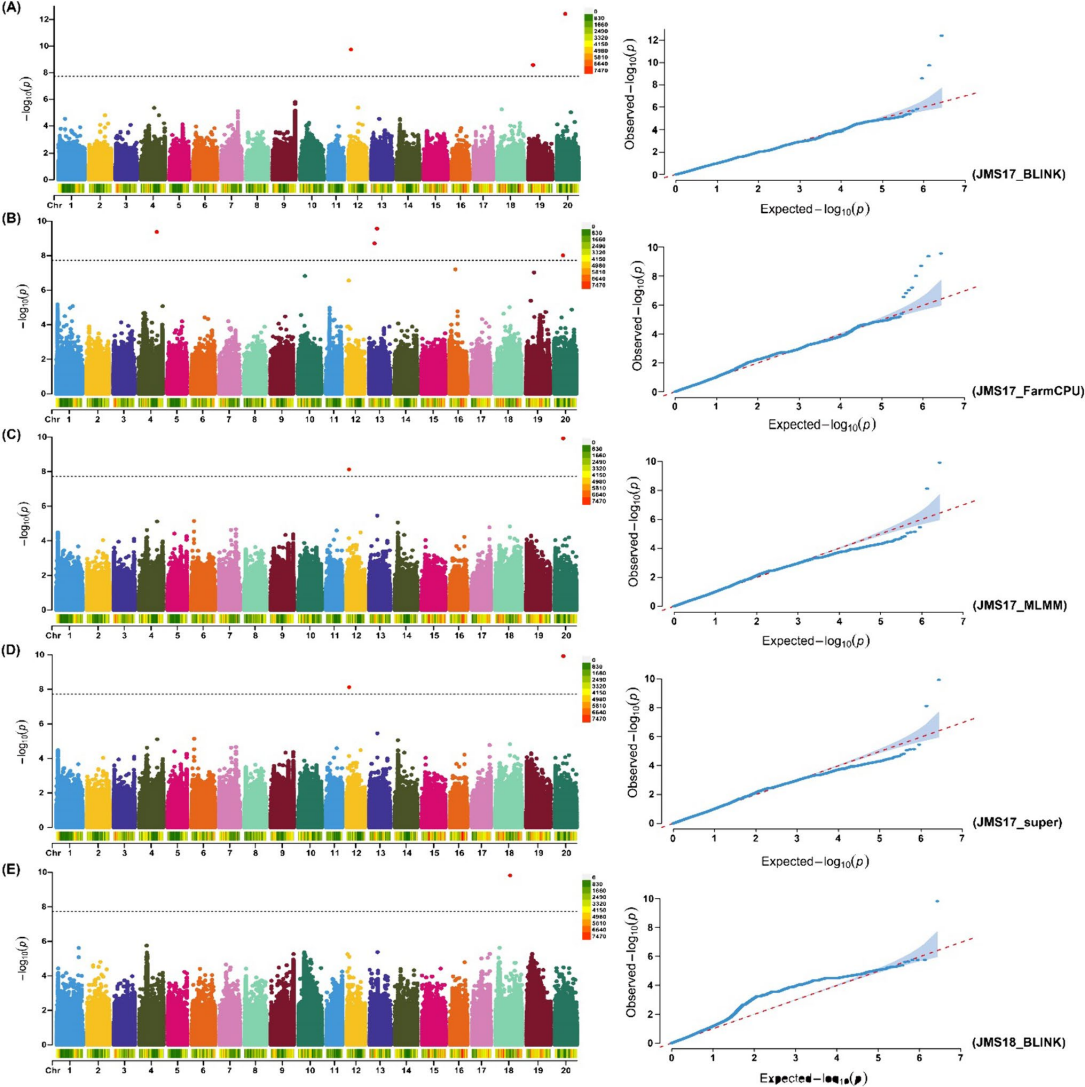
GWAS signals for branch number per plant evaluated across three environments (JMS17, JMS18 and JL20) and four GWAS models (MLMM, SUPER, FarmCPU and BLINK). Manhattan plot and quantile–quantile (Q–Q) plot for the GWAS for branch number per plant evaluated in different environments and GWAS models **A** JMS17_BLINK, **B** JMS17_FarmCPU, **C** JMS17_MLMM, **D** JMS17_super and **E** JMS18_BLINK. The black dotted lines on the Y-axis designate the significance threshold [− log_10_ (*P-value*) of > 7.73]. The numbers on the X-axis represent soybean chromosomes

**Fig. 4:**
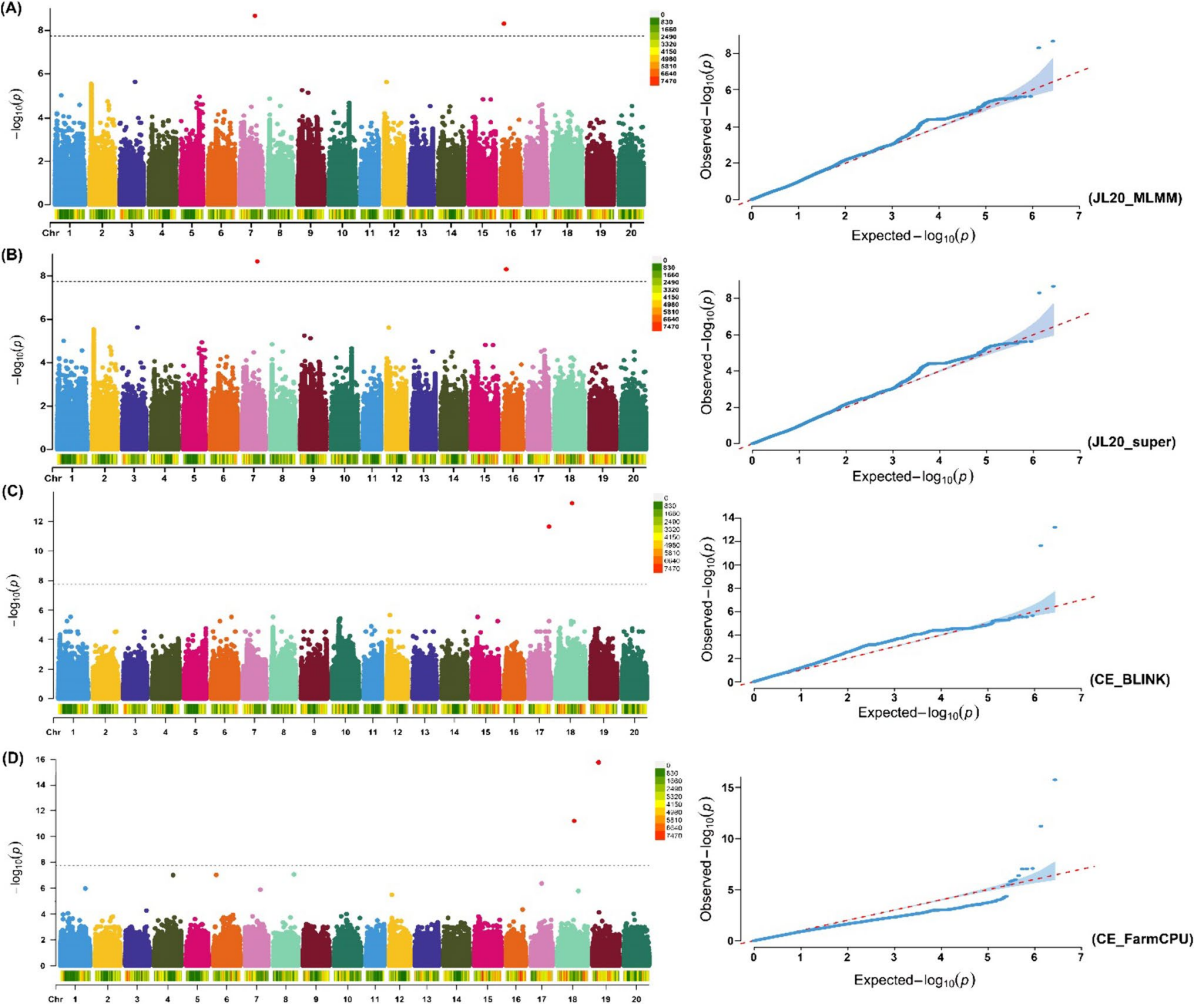
GWAS signals for branch number per plant evaluated across three environments (JMS17, JMS18 and JL20) plus combined environment (CE) and four GWAS models (MLMM, SUPER, FarmCPU and BLINK). Manhattan plot and quantile–quantile (Q–Q) plot for the GWAS for branch number per plant evaluated at different environment and GWAS models **A** JL20_MLMM, **B** JL20_super, **C** CE_BLINK and **D** CE_FarmCPU. The black dotted lines on the Y-axis designate the significance threshold [− log_10_ (*P-value*) of > 7.73]. The numbers on the X-axis represent soybean chromosomes

In the present study, we detected eleven SNPs that were significantly associated with branch number at -log_10_ (*P-value*) of 7.73 through five GWAS models across three different environments plus combined environments (Figs. 3 and 4; Table 2). The distribution of these significant SNPs showed their presence on nine chromosomes, viz., Chr.04, Chr.07, Chr.12, Chr.13, Chr.16, Chr.17, Chr.18, Chr.19 and Chr.20 out of total 20 soybean chromosomes. A maximum of two significant SNPs was detected on Chr.13 and Chr.19, followed by one SNP on each remaining seven chromosomes (Table 2). Moreover, of the eleven significant SNPs associated with branch number, one SNP, viz., Chr18_32068331, was consistently detected by all five GWAS models, viz., MLMM, super, BLINK, FarmCPU and 3VmrMLM. Both significant SNPs, viz., Chr12_5601871 and Chr20_18794801, were identified by four GWAS models except FarmCPU and 3VmrMLM, respectively (Table 2). The significant Chr07_29399935 was detected by three models, viz., MLMM, super and 3VmrMLM. However, three significant SNPs, viz., Chr13_17432336, Chr19_11321785 and Chr16_6096184, were detected by two GWAS models out of total five models used (Table 2). The remaining four SNPs such as Chr04_39125623, Chr13_12082492, Chr17_38943942, and Chr19_10711770 were detected by only one model out of total five models, viz., FarmCPU, FarmCPU, BLINK and BLINK, respectively. Furthermore, ten out of total eleven significant SNPs were detected by only one individual environment out of total three environments plus combined environment. The significant SNP Chr18_32068331 was detected in the two environments, viz., JMS 18 and combined environment. Hence, most of the significant SNPs detected for branch number showed variation in terms of their detection through different models and environments. This suggests that environmental variables were not consistent across the years in Jiamusi and Jilin locations, that is in agreement with the highly significant *E* and *G* × *E* interaction. However, out of the eleven SNPs, one SNP, viz., Chr18_32068331, showed significant *QTN* × *environment* (additive × environment) interaction (Table S6).

**Table 2:** Significant SNP markers associated with branch number across three environments

S. No.	Significant SNPs	Chr*	Environment*	Models/Methods	Position	*P*-value	− log *P*
1	Chr04_39125623	4	JMS17	FarmCPU	39,125,623	4.23E-10	9.373454
2	Chr07_29399935	7	JL20	MLMM, super, 3VmrMLM	29,399,935	2.13E-09	8.671305
3	Chr12_5601871	12	JMS17	MLMM, super, BLINK, 3VmrMLM	5,601,871	7.51E-09	8.124316
4	Chr13_12082492	13	JMS17	FarmCPU	12,082,492	1.95E-09	8.70886
5	Chr13_17432336	13	JMS17	FarmCPU, 3VmrMLM	17,432,336	2.72E-10	9.566225
6	Chr16_6096184	16	JL20	MLMM, super	6,096,184	4.92E-09	8.308236
7	Chr17_38943942	17	CE	BLINK	38,943,942	2.20E-12	11.65733
8	Chr18_32068331	18	JMS18, CE	MLMM, super, BLINK, FarmCPU, 3VmrMLM	32,068,331	1.52E-10	9.817911
9	Chr19_10711770	19	JMS17	BLINK	10,711,770	2.61E-09	8.583366
10	Chr19_11321785	19	CE	FarmCPU, 3VmrMLM	11,321,785	1.80E-16	15.74429
11	Chr20_18794801	20	JMS17	MLMM, super, FarmCPU, BLINK	18,794,801	3.73E-13	9.927639

^*^Chr (Chromosome), JMS17 (Jiamusi_*2017), JMS18 (Jiamusi*_*2018), JL20 (Jilin_2020), and CE (Combined environment)*

By considering the upstream and downstream distances within the linkage disequilibrium (LD) decay (± 202.4 kb) around the stable significant SNPs detected by multiple models and environments, we delineated the genomics regions within the physical distance of ± 202.4 kb around the stable significant SNPs, viz., Chr07_29399935, Chr12_5601871, Chr13_17432336, Chr16_6096184, Chr18_32068331, Chr19_11321785 and Chr20_18794801 present on the Chr.07, Chr.12, Chr.13, Chr.16, Chr.18, Chr.19 and Chr.20, respectively, as the QTLs. Hence, they were considered as seven stable QTLs regulating the branch number in soybean, viz., *qBN7*, *qBN12, qBN13, qBN16, qBN18, qBN19* and *qBN20*, respectively (Table 3). These QTLs/genomic regions showed consistency in multiple GWAS models and environments suggesting the stable nature of these QTLs governing branch number in soybean.

**Table 3:** Quantitative trait loci consistently linked with branch number across three different environments

S. No.	QTL	Chromosome	Position	Physical interval	Related QTLs	Reference
1	*qBN7*	Chr.07	29,399,935	29,197,535–29,602,335	No related QTL	Not available
2	*qBN12*	Chr.12	5,601,871	5,399,471–5,804,271	Branching 5–3	Shim et al. 2018
3	*qBN13*	Chr.13	17,432,336	17,229,936–17,634,736	No related QTL	Not available
4	*qBN16*	Chr.16	6,096,184	5,893,784–6,298,584	No related QTL	Not available
5	*qBN18*	Chr.18	32,068,331	31,865,931–32,270,731	No related QTL	Not available
6	*qBN19*	Chr.19	11,321,785	11,119,385–11,524,185	No related QTL	Not available
7	*qBN20*	Chr.20	18,794,801	18,592,401–18,997,201	No related QTL	Not available

### Haplotypes identification for branch number

**Fig. 5:**
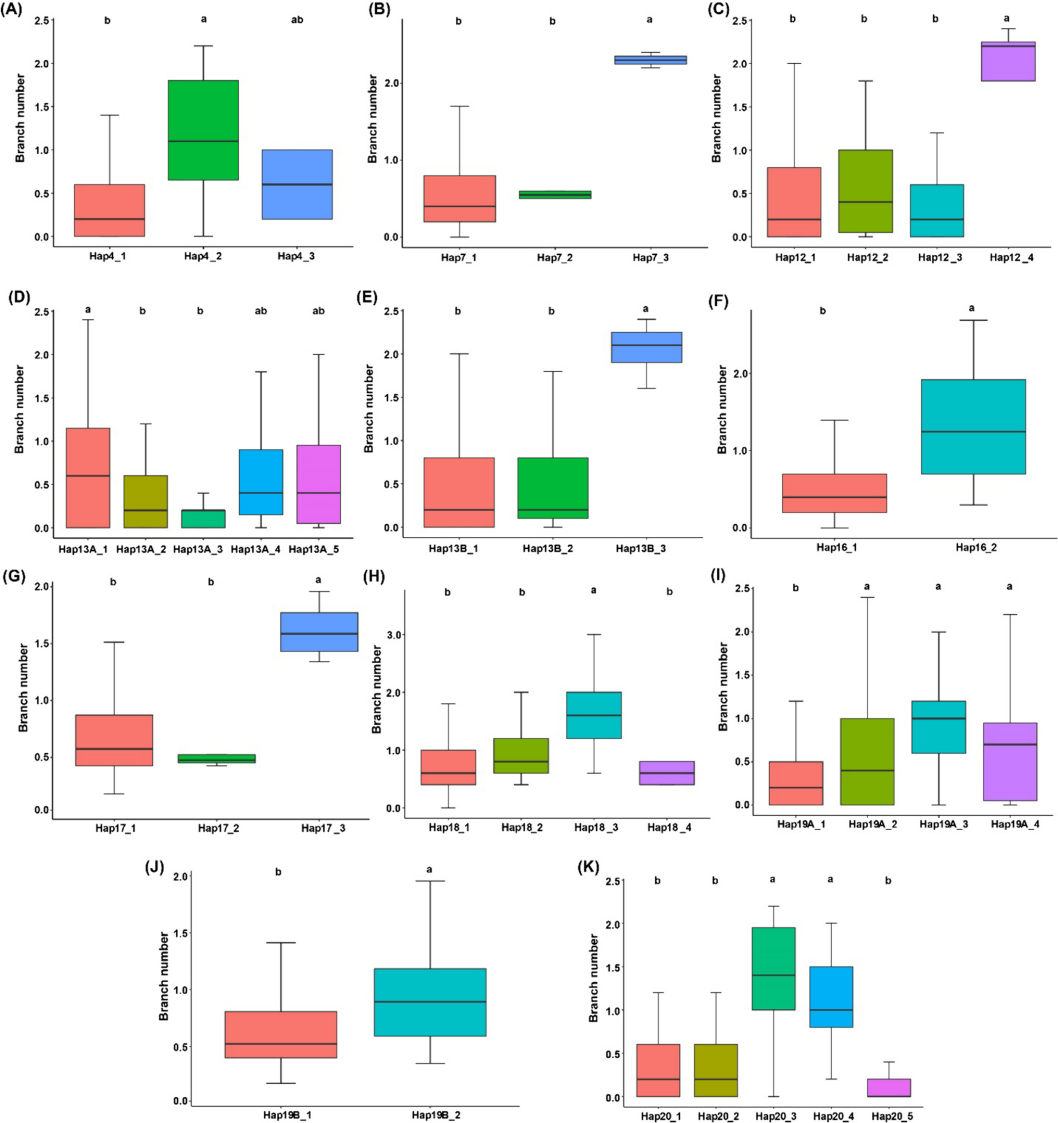
Haplotype allele analysis underlying eleven haplotype blocks, viz., Hap4, Hap7, Hap12, Hap13A, Hap13B, Hap16, Hap17, Hap18, Hap19A, Hap19B and Hap20. Haplotype boxplot revealed the predicted branch number values from combined environments. Grouping of genotypes and pairwise comparisons of genotypes was performed by using Tukey’s HSD test at *P* < 0.05. Common letters above the boxes represent the nonsignificant differences in branch number, whereas different letters represent significant differences

The closest adjacent SNPs present on the same chromosome in the upstream and downstream of the significant SNPs falling within the ± 0 kb distance form the haplotype blocks. In this study, we identified multiple SNPs on the Chr.04, Chr.07, Chr.12, Chr.13, Chr.13, Chr.16, Chr.18, Chr.19, Chr.19 and Chr.20 that are nearest to significant SNPs falling within the ± 0 kb physical distance, and thus they form haplotype blocks on their respective chromosomes (Fig. S1; Table S7). The four, four, four, five, four, two, six, five, three, six and five SNPs within the LD decay of ± 0 kb on the Chr.04, Chr.07, Chr.12, Chr.13, Chr.13, Chr.16, Chr17, Chr.18, Chr.19, Chr.19 and Chr.20, respectively, form eleven haplotype blocks, viz., Hap4, Hap7, Hap12, Hap13A, Hap13B, Hap16, Hap17, Hap18, Hap19A, Hap19B and Hap20, respectively (Fig. 5A–K).

The Hap4 underlies three haplotype alleles, viz., Hap4_1, Hap4_2 and Hap4_3 across the 200 soybean accessions, and these three showed significant differences in the regulation of branch number in soybean (Fig. 5A). Hap4_1 and Hap4_2 regulate the lower and higher branch number, respectively, and the remaining one haplotype (Hap4_3) controls the intermediate phenotype. Similarly, Hap7 possesses three haplotype alleles, and the branch number of the accessions varied significantly among the Hap7_1and Hap7_3 haplotypes, but the Hap7_1 and Hap7_2 showed nonsignificant difference in branch number regulation (Fig. 5B). For instance, Hap7_1 and Hap7_2 control the lower branch number, whereas the Hap7_3 controls the higher branch number. Moreover, Hap12 underlies four haplotype alleles, viz., Hap12_1, Hap12_2, Hap12_3 and Hap12_4 (Fig. 5C). The three haplotype alleles, viz., Hap12_1, Hap12_2 and Hap12_3, control lower branch number and showed nonsignificant difference; however, Hap12_4 regulates the higher branch number and showed significant different with the former three haplotype alleles. Haplotype block Hap13A possesses five haplotype alleles, viz., Hap13A_1, Hap13A_2, Hap13A_3, Hap13A_4 and Hap13A_5, respectively; Hap13A_2 and Hap13A_3 control lower branch number, and Hap13A_1 regulates higher branch number, whereas the Hap13A_4 and Hap13A_5 govern intermediate branch number (Fig. 5D). Moreover, the haplotype block Hap13B possesses three haplotype alleles, viz., Hap13B_1, Hap13B_2 and Hap13B_3; the Hap13B_1 and Hap13B_2 govern lower branch number, and the Hap13C_3 regulates the higher branch number (Fig. 5E). Haplotype block Hap16 possesses two haplotype alleles, viz., Hap16_1 and Hap16_2, regulating the significantly lowest and highest branch number, respectively (Fig. 5F). The three haplotypes alleles, viz., Hap17_1, Hap17_2 and Hap17_3, are possessed by the Hap17; the Hap17_1 and Hap17_3 regulate the contrasting phenotype of branch number, whereas Hap17_1 and Hap17_2 showed nonsignificant difference in the regulation of branch number (Fig. 5G). The Hap18 underlies four alleles, viz., Hap18_1, Hap18_2, Hap18_3 and Hap18_4; the Hap18_1, Hap18_2 and Hap18_4 control lowest branch number and showed nonsignificant difference, whereas the Hap18_3 controls significantly higher branch number relative to Hap18_1, Hap18_2 and Hap18_4 (Fig. 5H). Moreover, Hap19A also underlies four haplotype alleles, viz., Hap19A_1, Hap19A_2, Hap1A9_3 and Hap19A_4, regulating different phenotypes of branch number with significance difference among them (Fig. 5I). The Hap19B possesses two haplotype alleles, viz., Hap19B_1 and Hap19B_2, which showed significant difference in the regulation of branch number (Fig. 5J). Similarly, Hap20 contains five alleles, viz., Hap20_1, Hap20_2, Hap20_3, Hap20_4 and Hap20_5, that control varied phenotypes of branch number ranging from lowest to highest through the intermediate type (Fig. 5K). In conclusion, the haplotype alleles underlying the nine haplotype blocks regulate the different phenotypes of the branch number in soybean that varied from the lowest to highest through intermediate branching.

### Candidate gene identification

A total of 186 model genes were identified within the physical genomic interval of ± 202.4 kb of the eleven significant SNPs, viz., Chr04_39125623, Chr07_29399935, Chr12_5601871, Chr13_12082492, Chr13_17432336, Chr16_6096184, Chr17_38943942, Chr18_32068331, Chr19_10711770, Chr19_11321785 and Chr20_1879480, which consisted of 8, 9, 27, 13, 19, 37, 41, 3, 2, 18 and 9, respectively. Furthermore, based on the gene annotations, variant annotations (synonymous/nonsynonymous variant) and literature survey, we defined a total of 20 genes underlying the genomic interval of ± 202.4 kb of the eleven significant SNPs as possible candidates regulating branch number in soybean (Table 4 & Table S8). This includes one gene underlying the genomic interval (± 202.4 kb) of each significant SNPs, viz., Chr04_39125623, Chr07_29399935, Chr16_6096184, Chr18_32068331, Chr19_10711770, Chr19_11321785 and Chr20_18794801, five genes underlying Chr12_5601871, three genes underlying each Chr13_12082492, and Chr13_17432336, and two genes underlying Chr17_38943942 (Table 4). The selection of these candidate genes was based on gene function annotation such as genes governing axillary meristem and axillary bud growth, cell division, cell elongation, photoperiodism, flowering, vegetative to reproductive phase transition of meristem, growth hormones (auxin and cytokinin) and functions related to vegetative growth (Table 4). These 20 genes can be considered as putative candidates for controlling branch number in soybean.

**Table 4:** Potential candidate genes underlying identified QTLs, and Arabidopsis orthologs and gene annotations

S.No.	Model Gene	Arabidopsis ortholog	Gene function annotation
1	Glyma.04G159300	AT5G60910.1 (AGAMOUS-like 8)	Maintenance of inflorescence meristem identity; ovule development; positive regulation of flower development; regulation of anthocyanin biosynthetic process
2	Glyma.07G170500	AT5G44160.1(C2H2-like zinc finger protein)	Photoperiodism, flowering; positive regulation of transcription, DNA-dependent; regulation of timing of transition from vegetative to reproductive phase; response to nitrate
3	Glyma.12G073300	AT2G28550.3 (related to AP2.7)	Organ morphogenesis; abscisic acid stimulus; vegetative to reproductive phase transition of meristem
4	Glyma.12G073900	AT5G02810.1 (pseudo-response regulator 7)	Circadian rhythm; long-day photoperiodism, flowering; regulation of circadian rhythm; regulation of transcription, DNA-dependent
5	Glyma.12G074600	AT3G46290.1 (hercules receptor kinase 1)	Brassinosteroid mediated signaling pathway; post-embryonic development; regulation of unidimensional cell growth; response to brassinosteroid stimulus; unidimensional cell growth
6	Glyma.12G075300	AT5G02600.1 (Heavy metal transport/detoxification superfamily protein)	Flower development; phloem transport; root morphogenesis; sodium ion homeostasis
7	Glyma.12G074400	AT5G59990.1 (CCT motif family protein)	Regulation of flower development
8	Glyma.13G038500	AT5G15310.1 (myb domain protein 16)	Cell morphogenesis; response to auxin stimulus; response to ethylene stimulus; response to gibberellin stimulus; response to jasmonic acid stimulus; response to salicylic acid stimulus
9	Glyma.13G039300	AT2G30810.1 (Gibberellin-regulated family protein)	Response to gibberellin stimulus
10	Glyma.13G039600	AT2G30810.1(Gibberellin-regulated family protein)	Response to gibberellin stimulus
11	Glyma.13G072100	AT5G07990.1(Cytochrome P450 superfamily protein)	Anthocyanin-containing compound biosynthetic process; response to UV; response to UV-B; response to auxin stimulus; response to sucrose stimulus
12	Glyma.13G073400	AT5G06100.3 (myb domain protein 33)	Gibberellic acid mediated signaling pathway; gibberellin biosynthetic process; negative regulation of growth; pollen sperm cell differentiation
13	Glyma.13G072800	AT3G56580.2 (RING/U-box superfamily protein)	Protein ubiquitination; negative regulation of proline biosynthetic process
14	Glyma.16G060500	AT3G01780.1(ARM repeat superfamily protein)	Cellulose biosynthetic process; cytokinesis; pollen development
15	Glyma.17g233900	AT4G07410.1 (Transducin family protein /WD-40 repeat family protein)	Meristem maintenance; meristem growth; post-embryonic animal organ development; regulation of auxin polar transport
16	Glyma.17g235300	AT3G03990.1 (alpha/beta-Hydrolases superfamily protein)	secondary shoot formation; strigolactone biosynthetic process; cellular response to strigolactone;
17	Glyma.18G155000	AT3G18990.1 (AP2/B3-like transcriptional factor family protein)	Regulation of flower development; regulation of transcription, DNA-dependent; vernalization response
18	Glyma.19G057300	AT2G23180.1 (cytochrome P450, family 96, subfamily A, polypeptide 1)	Oxidation–reduction process
19	Glyma.19g057900	AT1G05830.1 (trithorax-like protein 2) |	Vegetative to reproductive phase transition of meristem; histone H3-K4 dimethylation; histone H3-K4 methylation;
20	Glyma.20G060400	AT3G07650.4 (CONSTANS-like 9)	Circadian rhythm; negative regulation of long-day photoperiodism, flowering

**Fig. 6:**
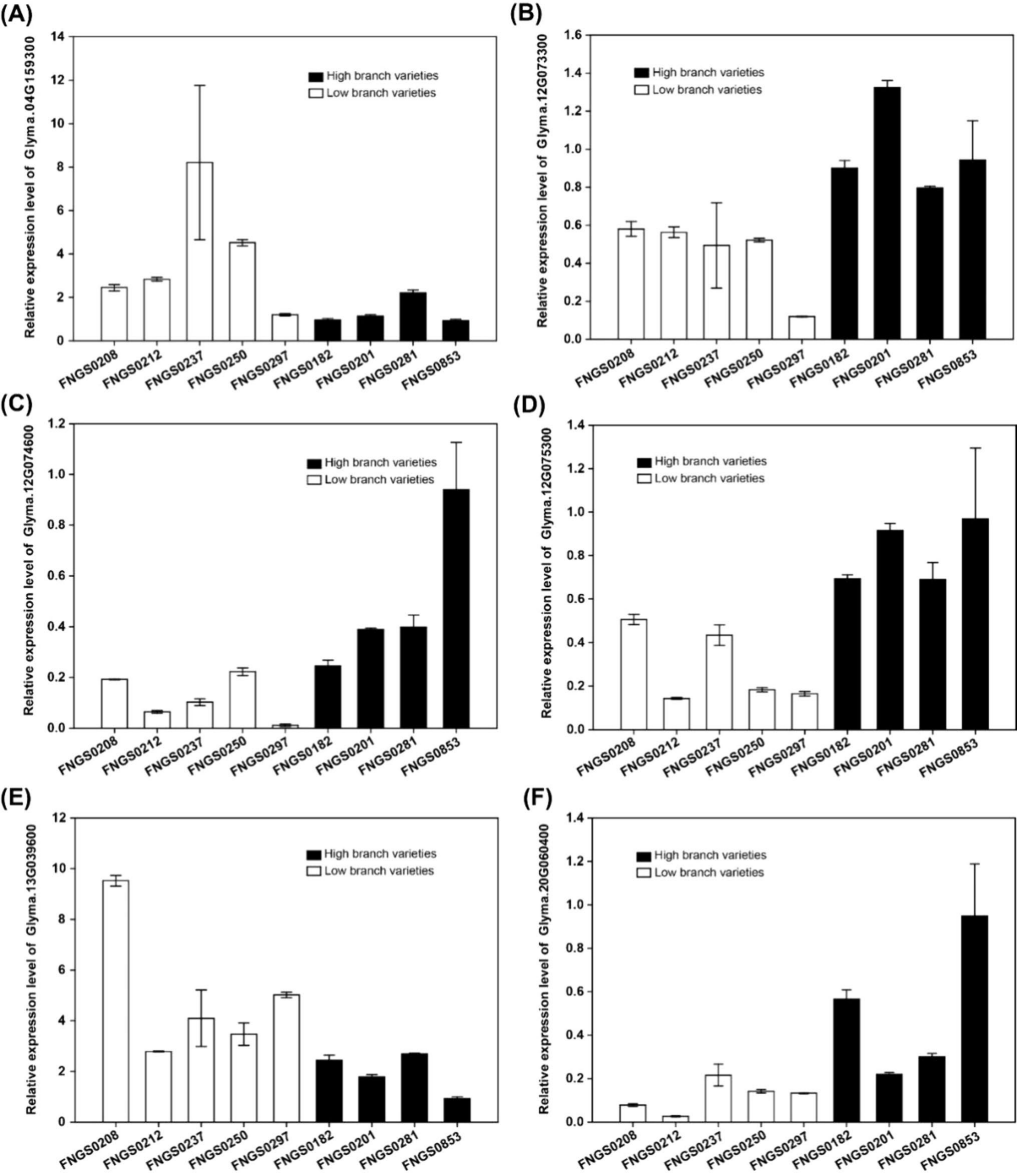
The qRT-PCR analysis of the six candidate genes, viz., **A** *Glyma.04G159300*, **B** *Glyma.12G073300*, **C** *Glyma.12G074600*, **D** *Glyma.12G075300*, **E** *Glyma.13G039600* and **F** *Glyma.20G060400,* showing differential expression patterns across soybean cultivars with contrasting branch number phenotype. The white bars represent the cultivars with low branch number, and black bars represent the cultivars with high branch number. For the comparison of gene expression among the soybean cultivars, the FNG0853 cultivar was set as the control

The above 20 putative candidate genes were further subjected to qRT-PCR analysis by using five soybean cultivars with low branch number, and four cultivars with high branch number. Out of the 20 genes, the six genes, viz., *Glyma.04G159300*, *Glyma.12G073300*, *Glyma.12G074600*, *Glyma.12G075300*, *Glyma.13G039600* and *Glyma.20G060400*, showed significant differential expression patterns between the contrasting soybean genotypes (Fig. 6). The remaining 14 genes showed nonsignificant differential expression patterns between contrasting genotypes. Hence, these six genes can be considered as the potential candidate genes regulating branch number in soybean. However, further functional validation of these genes is needed to determine their exact role in regulating branch number in soybean.

## Discussion

The branch number in soybean plants is a major determinant of soybean architecture and seed yield (Shim et al. 2019). This trait is regulated by complex processes involving axillary meristem initiation followed by spatial–temporal regulation of axillary bud outgrowth (Tanaka et al. 2015). It is an important objective for soybean breeders to produce cultivars with desirable branch numbers and improved seed yields. Hence, intensive efforts are needed to characterize the soybean germplasm in relation to branch number to address the yield gain needs. Breeders have made efforts to select desirable branch numbers in soybean using the principles and methods of conventional breeding (Liu et al. 2020; Rani and Kumar 2022). However, branch number is a complex quantitative trait which is highly influenced by the environment; thus, conventional efforts have not met breeding demands (Shim et al. 2019). In this regard, molecular breeding has emerged as a potential approach for breeding improved soybean cultivars with higher precision and accuracy (Bhat et al. 2022b). However, in molecular breeding, it is important first to know the genetic basis of the traits of interest, such as branch number, and use the detected QTLs/genes/haplotypes associated with such traits in soybean breeding. The present study used the integrated approach of GWAS and haplotype and candidate gene analyses to elucidate the detailed gene architecture associated with branch number in soybean.

In the present study, a GWAS panel of 200 soybean accessions was evaluated for branch number at two locations, the Jiamusi and Jilin experimental farms, representing total of three environments viz., JMS17, JMS18 and JL20 plus combined environment. Our results revealed that the soybean accessions possessed significant genotypic variance, which is the basis for modulating branch number in soybean. These results are aligned with previous findings (Borah et al. 2018; Shim et al. 2019). The effect of genotype (*G*), environment (*E*) and *G* × *E* interactions on the branch number was highly significant. This suggests that genotypes of the GWAS panel possess considerable genetic diversity; in addition, the environmental variables were different across the different environments, which in turn contributed to the different expression of the branch number of the same set of accessions grown in the different environments. Higher broad-sense heritability revealed by the branch number suggests that the same set of soybean accessions will show the same performance if grown in the same environmental conditions. These findings are similar to those previously reported by different studies that investigated branch number in soybean. (Shim et al. 2017; Guang et al. 2017; Borah et al. 2018). In addition, highly significant *G*, *E* and *G* × *E* interactions revealed by branch number indicate its complex inheritance pattern, similar to what was observed in earlier studies (He et al. 2014; Shim et al. 2019).

Limited studies have been performed to elucidate the genetic basis of branch number in soybean (Chen et al. 2007; Li et al. 2008; Sayama et al. 2010; Yao et al. 2015; Shim et al. 2017). In addition, these studies have used the conventional approach of QTL mapping and low-density linkage maps that identified QTLs at low resolution, which in turn hindered their use in soybean breeding (Bhat et al. 2021). However, recent advances in sequencing, high-density marker genotyping and GWAS approaches have allowed the identification of marker‒trait associations and underlying genes at higher resolution for crop traits (Alqudah et al. 2020). Many previous studies have used the GWAS approach to elucidate the genetic makeup of different traits in soybean, including branch number (Shim et al. 2019; Zhang et al. 2021). In this context, we used GWAS together with high-density SNP markers to unravel the genetic basis of branch number in soybean. We identified a total of eleven SNPs significantly linked with branch number at the -log_10_ (*P-value*) of > 7.73 across the three different environments plus combined environment and five GWAS models. These SNPs were distributed across nine soybean chromosomes out of total 20 soybean chromosomes, suggesting the complex polygenic nature of branch number in soybean. These results are similar to those previously reported by many authors investigated branch number in soybean (He et al. 2014; Shim et al. 2019; Zhang et al. 2021). Moreover, our results showed considerable variation in the detection of the significant SNPs by five GWAS models, i.e., some SNPs were detected by one model. For example, the SNP, viz., Chr18_32068331, was detected through all the five GWAS models, while in contrast, four significant SNPs among the total eleven were identified through only one GWAS model. Similar results were previously reported in different studies, such as in soybean (Bhat et al. 2022b), maize (Kaler et al. 2020) and wheat (Merrick et al. 2022). This can be explained by the fact that different GWAS models are based on different hypotheses involving varied QTL effect distribution characteristics (Bhat et al. 2021). In addition, the significant SNPs also showed variation in terms of their detection in different environments. This suggests that environmental variables did not show consistency across the years at the Jiamusi and Jilin locations, and this is aligned with the highly significant *E* and *G* × *E* interaction identified in the present study. Moreover, one SNP, viz., Chr18_32068331, out of total eleven significant SNPs detected in the present study showed significant *QTN* × *environment* (additive × environment) interaction.

Importantly, the significant SNPs identified by multiple GWAS models and environments on the Chr.07, Chr.12, Chr.13, Chr.16, Chr.18, Chr.19 and Chr.20 were considered as stable MTAs. The genomic regions (± 202.4 kb) flanking these significant SNPs were referred to as QTLs related to branch number. These QTLs/genomic regions represent stable genetic elements regulating branch number in soybean. Among these QTLs, *qBN12* on Chr.12 associated with branch number was previously reported in the physical interval of 5,415,879 and 7,533,328 bp by Shim et al. (2017), and our results showed that the physical interval falls in the same genomic region. Therefore, *qBN12* might belong to the same Branching 5–3 QTL, as previously reported by Shim et al. (2017). Moreover, our study showed that the physical interval of Branching 5–3 was considerably narrowed down. However, no QTL has been identified to date that falls within the physical interval of *qBN7*, *qBN13, qBN16, qBN18, qBN19* and *qBN20*. Hence, these six QTLs can be regarded as novel QTLs for branch number identified in the current study. The results of the present study considerably narrowed down the physical interval for the previously reported QTL, which indicates the higher resolution of the GWAS approach compared to the linkage mapping approach. The QTL mapping approach has used the biparental mapping population and low-density markers, which are responsible for the low resolution of the previously reported QTLs (Kraakman et al. 2004). Therefore, the high resolution of the GWAS analysis in the identification of stable QTLs across multiple models and environments will facilitate their effective utilization in MAB programs for breeding soybean cultivars with desirable branch numbers.

The potential of GWAS in gene identification for complex traits has been well recognized by the research community. However, mostly biallelic SNP markers have been used for the GWAS analysis in soybean, which has resulted in the failure to detect superior and rare alleles regulating desirable phenotypes for crop traits (Bhat et al. 2021). Hence, crop researchers were always looking for the multiallelic markers that can capture the epistatic variation and superior/rare alleles in the diverse crop germplasm. In this context, the recent emergence of the haplotype markers has fulfilled this demand that are multiallelic with the huge ability to fix superior/rare alleles and epistatic variation (Luján Basile et al. 2019). Therefore, the use of haplotype markers in crop breeding will prevent the loss of important genetic variation and make it available for crop improvement (Bhat et al. 2021). Recently the superior haplotypes have been identified in soybean for the plant height (Bhat et al. 2022a; Yu et al. 2023), yield-related traits (Bhat et al. 2022b) and salt tolerance (Patil et al. 2016). The superior haplotypes were also identified for different traits in other crops such as grain quality traits of rice (Wang et al. 2017) and drought tolerance in pigeonpea (Sinha et al. 2020). Genetic mechanisms, such as mutation, recombination and selection, are the major components regulating the haplotype variation in the crop germplasm (Zaitlen et al. 2005). Precise identification of haplotypes for the crop traits with their utilization in the marker-assisted breeding will harness the enormous potential of genetic diversity in crop improvement (Sinha et al. 2020). In the current study, we detected more than two haplotype alleles varying from two to five underlying the eleven identified haplotype blocks on eight different chromosomes. These haplotypes alleles of each block govern the different phenotypes of branch number, thus providing an opportunity to modify soybean branch number in multiple ways as per breeder’s requirement. Deployment of these haplotypes in soybean breeding will allow us to produce soybean cultivars with the desired branch number and plant type, which in turn will have a great impact on soybean yield and quality.

It is the ultimate goal of crop researchers to identify the actual candidate genes underlying the major genomic regions regulating the trait of interest (Ganie and Ahammed 2021; Ganie et al. 2021). Proper functional verification of the identified candidate genes determines their actual use in crop breeding. Branch number has remained underestimated as far as the elucidation of the genetic basis is concerned, and very few genes for branch number in soybean have been characterized (Borah et al. 2018; Shim et al. 2019). Based on the in silico analysis, our study identified 20 candidate genes underlying the genomic regions of ± 202.4 kb around the eleven identified significant SNPs. Among these 20 candidate genes, the two significant SNPs viz., Chr12_5601871 and Chr13_17432336 were present within the exonic region of the candidate gene, viz., *Glyma.12G074400* and *Glyma.13G072800*, respectively, producing the synonymous and nonsynonymous mutation. Thus, these two genes were also selected as candidate genes for branch number. The remaining 18 candidate genes were defined as possible candidates regulating branch number in soybean, because these genes regulate many gene functions, such as those related to regulation of axillary meristem and axillary bud growth, meristem growth, cell division, cell elongation, photoperiodism, flowering, meristem vegetative to reproductive phase transition, growth hormones (auxin and cytokinin) and functions related to vegetative growth that are involved in shoot branching. Hence, these 18 candidate genes selected in the present study possess at least one or more gene functions related to the above functions/processes. For example, the gene functions of *Glyma.13g038500*, *Glyma.13g072100* and *Glyma.17g233900* are related to auxin signalling, transport and response, and *Glyma.16g060500* is involved in the cytokinin signalling. It is well documented that plant hormones, viz., auxin and cytokinin, control shoot branching in flowering plants (Coudert et al. 2015). It has been known for many decades that auxin inhibits the activation of axillary buds and hence shoot branching, while cytokinin has the opposite effect (Muller and Leyser, 2011). Auxin moves down the main shoot of the plant to prevent new branches from forming. This movement is controlled by PIN proteins and several other families of proteins. On the other hand, cytokinin promotes the growth of new branches. Moreover, the functions of the genes *Glyma.07g170500*, *Glyma.12g073300* and *Glyma.19g057900* are related to the meristem vegetative to reproductive phase transition. Similarly, the functions of the genes *Glyma.04g159300*, *Glyma.07g170500*, *Glyma.12g073900*, *Glyma.12g075300*, *Glyma.12G074400*, *Glyma.18g155000* and *Glyma.20g060400* are involved in flowering development and regulation. The regulation of flowering as well as the plant vegetative to reproductive phase transition controls branching in crop plants because branches develop from the leaf axils at each unelongated node of the main shoot during vegetative growth (Krishnan et al. 2011). The *Glyma.07g170500*, *Glyma.12g073900* and *Glyma.20g060400* are involved in the regulation of circadian rhythm and photoperiodism. The circadian system regulates the effects of photoperiod on the transition from vegetative to reproductive growth and flowering timing in plants (Imaizumi and Kay, 2006). *Glyma.13g073400*, *Glyma.13g039300* and *Glyma.13g039600* are related to the gibberellic acid-mediated signaling pathway and response to gibberellin stimulus. Shan et al. (2021) documented that cell division and elongation promoted by plant growth hormones such as gibberellins (GAs) increase stem/branch elongation in soybean. The *Glyma.12g074600* function is related to the brassinosteroid-mediated signaling pathway, and Xia et al. (2021) demonstrated that brassinosteroid signaling integrates multiple pathways that control shoot branching. They also revealed that local brassinosteroid signaling in axillary buds is a potential target for shaping plant architecture. *Glyma.19g057300* belongs to cytochrome P450; and specifically, they have been documented to regulate shoot patterning and flower development by controlling the hormone homeostasis (Distéfano et al. 2021). For example, strigolactones are important hormone for shoot branching, and the carotenoid is the precursor for the synthesis of strigolactones, and carotenoid precursor is carlactone. In the strigolactones biosynthesis pathway, MAX1 encodes a CYP711A1 that catalyzes the conversion of carlactone into carlactonoic acid (Abe et al., 2014). It has been observed that MAX1 mutant revealed abnormally abundant branches as well as abnormal expression pattern of auxin carrier’s influx and efflux in the stems. The gene function of the *Glyma.17g235300* is involved in the strigolactone biosynthetic process. Furthermore, the qRT-PCR analysis revealed that six genes, viz., *Glyma.04G159300*, *Glyma.12G073300*, *Glyma.12G074600*, *Glyma.12G075300*, *Glyma.13G039600* and *Glyma.20G060400*, from the total 20 candidate genes identified by in silico analysis showed differential expression patterns between soybean genotypes possessing contrasting branch number phenotype. Hence, these six genes will be considered as the potential candidate genes regulating branch number in soybean. However, before these candidate genes can be deployed in marker-assisted breeding for modulating branch number in soybean, their function must be verified by using gene validation tests such as knockout or overexpression experiments. After the function of these genes are verified, they can be used directly for the breeding of desirable branch numbers in soybean.

## Conclusion

In the current study, we used a combined approach of GWAS, haplotype analysis and candidate genes to unravel the genetic basis of branch number in soybean. A total of eleven SNPs significantly linked with branch number were identified by GWAS, and seven stable QTL regions, viz., *qBN7*, *qBN12, qBN13, qBN16, qBN18, qBN19 and qBN20,* were also identified. Among these QTLs, six QTLs (*qBN7*, *qBN13, qBN16, qBN18, qBN19 and qBN20*) were novel, whereas the remaining one QTL (*qBN12*) has been previously reported. In addition, in silico analysis prioritized the 20 genes underlying the genomic regions of ± 202.4 kb around the eleven identified significant SNPs as putative candidates. Out of them six genes showed differential expression patterns among the soybean genotypes with contrasting branch number phenotype, thus were considered as potential candidate genes regulating branch number in soybean. Two to five haplotype alleles detected across eleven haplotype blocks controlled diverse phenotypic values of branch number ranging from the lowest to highest through the intermediate branching type. Overall, superior haplotypes, stable QTLs and candidate genes for branch number detected in the current study can serve as potential resources for modulating branch number and seed yield in soybean. Using different genetic backgrounds for the validation of QTLs and haplotypes can allow their direct utilization in marker-assisted breeding in soybean. In addition, proper validation of candidate genes using overexpression or gene knockout studies can allow their direct use in the development of soybean cultivars with desirable branch numbers. Hence, the present study provides a critical analysis of diverse soybean germplasm and identifies novel genomic resources for soybean improvement.

### Supplementary Information

Below is the link to the electronic supplementary material.

Supplementary file1 (DOCX 444 KB)

## Data Availability

Resequencing data generated in this study has been deposited in the VCF format at Genome Variation Map (GVM) database in BIG Data Center (http://bigd.big.ac.cn/gvm) under the accession number: GVM00044.
