# Supplementary material for: Identification of superior and rare haplotypes to optimize branch number in soybean
Source: Theor Appl Genet. 2024 Apr 3;137(4):93. doi: 10.1007/s00122-024-04596-y (PMC10991007; doi:10.1007/s00122-024-04596-y)
Supplement: Supplementary file 1 — Supplementary file1 (DOCX 444 KB) [file 122_2024_4596_MOESM1_ESM.docx]

**
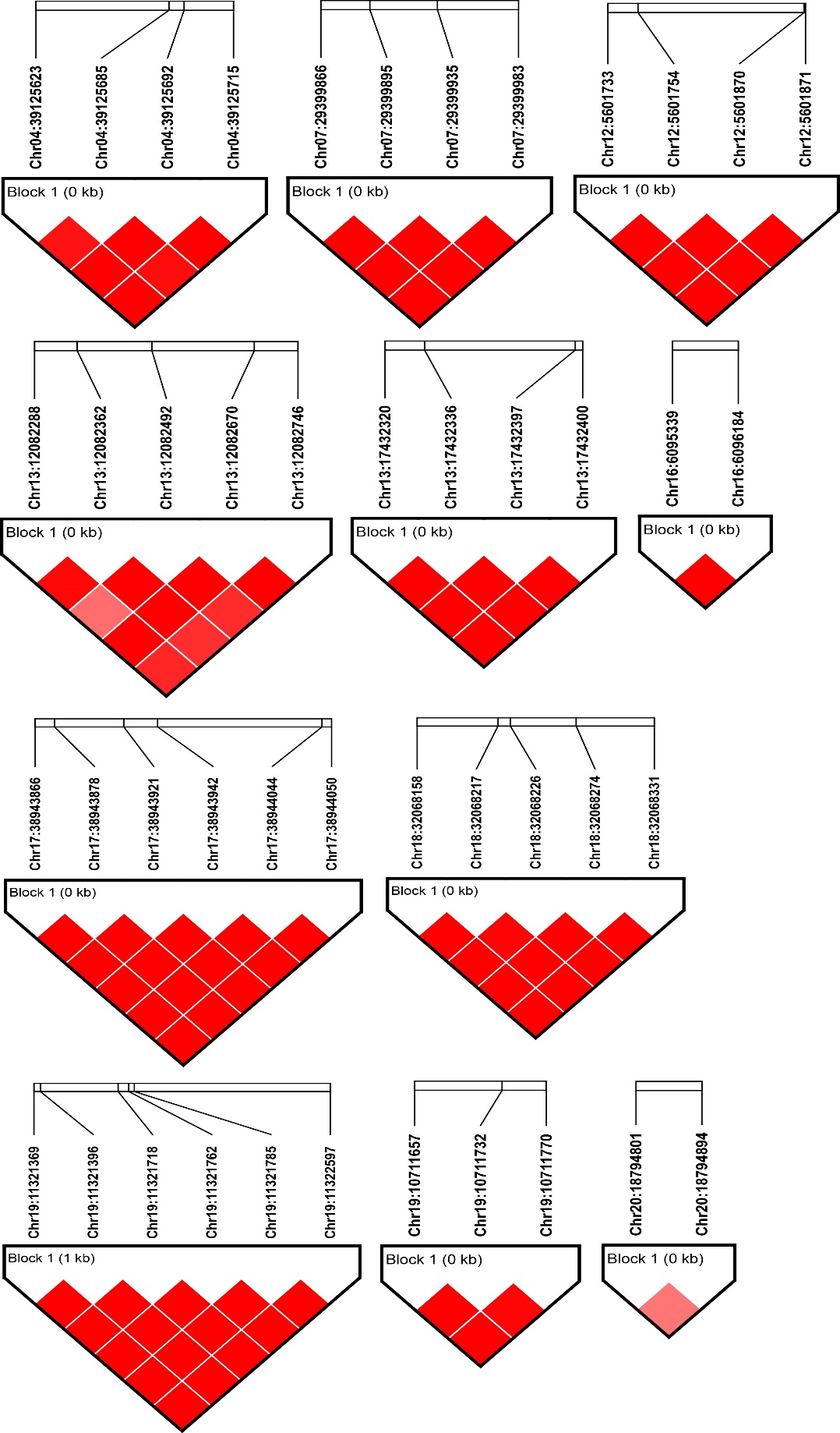
**

**Fig. S1**: Eleven haplotype blocks viz., Hap4, Hap7, Hap12, Hap13A, Hap13B, Hap16, Hap17, Hap18, Hap19A, Hap19B and Hap20 identified on the Chr.04, Chr.07, Chr.12, Chr.13, Chr.13, Chr.16, Chr.17, Chr.18, Chr.19, Chr.19 and Chr.20, respectively.

**Table S1:** Grouping of the 200 soybean accessions of GWAS panel into five accumulated temperature zones viz., I, II, III, IV & V (in each zone the temperature remains constant).

| Name of genotype | Division of accumulated temperature zone( Group_I-V) | Geographic origin |
| --- | --- | --- |
| FNGS0157 | I | Beian, Heilongjiang, China |
| FNGS0158 | I | Beian, Heilongjiang, China |
| FNGS0159 | I | Beian, Heilongjiang, China |
| FNGS0160 | I | Beian, Heilongjiang, China |
| FNGS0161 | I | Hegang, Heilongjiang, China |
| FNGS0162 | I | Beian, Heilongjiang, China |
| FNGS0163 | I | Beian, Heilongjiang, China |
| FNGS0164 | I | Heihe, Heilongjiang, China |
| FNGS0165 | I | Shuangyashan, Heilongjiang, China |
| FNGS0166 | I | Shuangyashan, Heilongjiang, China |
| FNGS0167 | I | Beian, Heilongjiang, China |
| FNGS0168 | I | Beian, Heilongjiang, China |
| FNGS0169 | I | Beian, Heilongjiang, China |
| FNGS0170 | I | Beian, Heilongjiang, China |
| FNGS0171 | I | Heihe, Heilongjiang, China |
| FNGS0172 | II | Hulunbeier, Inner Mongolia,China |
| FNGS0173 | IV | Harbin, Heilongjiang,China |
| FNGS0174 | V | Harbin, Heilongjiang,China |
| FNGS0175 | II | Harbin, Heilongjiang,China |
| FNGS0176 | I | Harbin, Heilongjiang,China |
| FNGS0177 | II | Harbin, Heilongjiang,China |
| FNGS0178 | I | Harbin, Heilongjiang,China |
| FNGS0179 | III | Harbin, Heilongjiang,China |
| FNGS0180 | IV | Harbin, Heilongjiang,China |
| FNGS0181 | III | Harbin, Heilongjiang,China |
| FNGS0182 | II | Harbin, Heilongjiang,China |
| FNGS0183 | I | Harbin, Heilongjiang,China |
| FNGS0184 | I | Harbin, Heilongjiang,China |
| FNGS0185 | II | Harbin, Heilongjiang |
| FNGS0187 | I | Beian, Heilongjiang, China |
| FNGS0188 | I | Beian, Heilongjiang, China |
| FNGS0189 | I | Beian, Heilongjiang, China |
| FNGS0190 | III | Jiamusi, Heilongjiang,China |
| FNGS0191 | II | Jiamusi, Heilongjiang,China |
| FNGS0192 | II | Jiamusi, Heilongjiang,China |
| FNGS0193 | I | Jiamusi, Heilongjiang,China |
| FNGS0194 | I | Jiamusi, Heilongjiang,China |
| FNGS0195 | II | Jiamusi, Heilongjiang,China |
| FNGS0196 | I | Jiamusi, Heilongjiang,China |
| FNGS0197 | I | Jiamusi, Heilongjiang,China |
| FNGS0198 | I | Jiamusi, Heilongjiang,China |
| FNGS0199 | I | Jiamusi, Heilongjiang,China |
| FNGS0200 | II | Jiamusi, Heilongjiang,China |
| FNGS0201 | II | Jiamusi, Heilongjiang,China |
| FNGS0202 | II | Jiamusi, Heilongjiang,China |
| FNGS0203 | II | Jiamusi, Heilongjiang,China |
| FNGS0204 | II | Jiamusi, Heilongjiang,China |
| FNGS0205 | II | Jiamusi, Heilongjiang,China |
| FNGS0206 | II | Jiamusi, Heilongjiang,China |
| FNGS0207 | II | Jiamusi, Heilongjiang,China |
| FNGS0208 | I | Jiamusi, Heilongjiang,China |
| FNGS0209 | III | Jiamusi, Heilongjiang,China |
| FNGS0211 | I | Jiamusi, Heilongjiang,China |
| FNGS0212 | II | Jiamusi, Heilongjiang,China |
| FNGS0213 | V | Jiamusi, Heilongjiang,China |
| FNGS0214 | I | Jiamusi, Heilongjiang,China |
| FNGS0215 | II | Jiamusi, Heilongjiang,China |
| FNGS0216 | II | Jiamusi, Heilongjiang,China |
| FNGS0217 | II | Jiamusi, Heilongjiang,China |
| FNGS0218 | I | Jiamusi, Heilongjiang,China |
| FNGS0219 | I | Jiamusi, Heilongjiang,China |
| FNGS0220 | II | Jiamusi, Heilongjiang, China |
| FNGS0221 | I | Beian, Heilongjiang, China |
| FNGS0222 | I | Heihe, Heilongjiang, China |
| FNGS0223 | I | Heihe, Heilongjiang, China |
| FNGS0224 | I | Heihe, Heilongjiang, China |
| FNGS0225 | I | Heihe, Heilongjiang, China |
| FNGS0226 | I | Heihe, Heilongjiang, China |
| FNGS0227 | I | Heihe, Heilongjiang, China |
| FNGS0228 | I | Heihe, Heilongjiang, China |
| FNGS0229 | I | Heihe, Heilongjiang, China |
| FNGS0230 | I | Heihe, Heilongjiang, China |
| FNGS0231 | I | Heihe, Heilongjiang, China |
| FNGS0232 | I | Heihe, Heilongjiang, China |
| FNGS0233 | I | Heihe, Heilongjiang, China |
| FNGS0234 | I | Heihe, Heilongjiang, China |
| FNGS0235 | II | Harbin, Heilongjiang, China |
| FNGS0236 | III | Harbin, Heilongjiang, China |
| FNGS0237 | II | Harbin, Heilongjiang, China |
| FNGS0238 | III | Harbin, Heilongjiang, China |
| FNGS0239 | II | Harbin, Heilongjiang, China |
| FNGS0240 | II | Harbin, Heilongjiang, China |
| FNGS0241 | II | Harbin, Heilongjiang, China |
| FNGS0242 | IV | Harbin, Heilongjiang, China |
| FNGS0243 | IV | Harbin, Heilongjiang, China |
| FNGS0244 | II | Harbin, Heilongjiang, China |
| FNGS0245 | V | Harbin, Heilongjiang, China |
| FNGS0246 | II | Harbin, Heilongjiang, China |
| FNGS0247 | III | Harbin, Heilongjiang, China |
| FNGS0248 | IV | Harbin, Heilongjiang, China |
| FNGS0249 | II | Harbin, Heilongjiang, China |
| FNGS0250 | II | Harbin, Heilongjiang, China |
| FNGS0251 | II | Harbin, Heilongjiang, China |
| FNGS0252 | II | Harbin, Heilongjiang, China |
| FNGS0253 | III | Harbin, Heilongjiang, China |
| FNGS0254 | I | Beian, Heilongjiang, China |
| FNGS0255 | I | Beian, Heilongjiang, China |
| FNGS0256 | I | Beian, Heilongjiang, China |
| FNGS0257 | I | Beian, Heilongjiang, China |
| FNGS0258 | II | Jiamusi, Heilongjiang, China |
| FNGS0259 | II | Harbin, Heilongjiang, China |
| FNGS0260 | II | Harbin, Heilongjiang, China |
| FNGS0261 | I | Heihe, Heilongjiang, China |
| FNGS0262 | I | Heihe, Heilongjiang, China |
| FNGS0263 | III | Qiqihar, Heilongjiang, China |
| FNGS0264 | II | Harbin, Heilongjiang, China |
| FNGS0265 | II | Harbin, Heilongjiang, China |
| FNGS0266 | II | Harbin, Heilongjiang, China |
| FNGS0267 | II | Harbin, Heilongjiang, China |
| FNGS0268 | III | Harbin, Heilongjiang, China |
| FNGS0269 | II | Harbin, Heilongjiang, China |
| FNGS0270 | I | Harbin, Heilongjiang, China |
| FNGS0271 | I | Beian, Heilongjiang, China |
| FNGS0272 | II | Daqing, Heilongjiang, China |
| FNGS0273 | II | Daqing, Heilongjiang, China |
| FNGS0274 | II | Daqing, Heilongjiang, China |
| FNGS0275 | II | Daqing, Heilongjiang, China |
| FNGS0276 | I | Hulunbeier, Heilongjiang, China |
| FNGS0277 | I | Hulunbeier, Heilongjiang, China |
| FNGS0278 | I | Nenjiang, Heilongjiang, China |
| FNGS0279 | IV | Binxian, Heilongjiang, China |
| FNGS0280 | I | Heihe, Heilongjiang, China |
| FNGS0281 | I | Suihua, Heilongjiang, China |
| FNGS0282 | III | Suihua, Heilongjiang, China |
| FNGS0283 | III | Suihua, Heilongjiang, China |
| FNGS0284 | II | Suihua, Heilongjiang, China |
| FNGS0285 | II | Suihua, Heilongjiang, China |
| FNGS0286 | I | Suihua, Heilongjiang, China |
| FNGS0287 | II | Suihua, Heilongjiang, China |
| FNGS0288 | III | Suihua, Heilongjiang, China |
| FNGS0289 | II | Suihua, Heilongjiang, China |
| FNGS0290 | III | Suihua, Heilongjiang, China |
| FNGS0291 | I | Suihua, Heilongjiang, China |
| FNGS0292 | I | Suihua, Heilongjiang, China |
| FNGS0293 | I | Suihua, Heilongjiang, China |
| FNGS0294 | I | Suihua, Heilongjiang, China |
| FNGS0295 | II | Suihua, Heilongjiang, China |
| FNGS0296 | II | Suihua, Heilongjiang, China |
| FNGS0297 | I | Suihua, Heilongjiang, China |
| FNGS0298 | III | Suihua, Heilongjiang, China |
| FNGS0299 | III | Suihua, Heilongjiang, China |
| FNGS0300 | II | Suihua, Heilongjiang, China |
| FNGS0301 | I | Suihua, Heilongjiang, China |
| FNGS0302 | IV | Changchun, Jilin,China |
| FNGS0303 | V | Changchun, Jilin,China |
| FNGS0304 | V | Changchun, Jilin,China |
| FNGS0305 | V | Changchun, Jilin,China |
| FNGS0306 | V | Changchun, Jilin,China |
| FNGS0307 | V | Changchun, Jilin,China |
| FNGS0308 | V | Changchun, Jilin,China |
| FNGS0309 | V | Changchun, Jilin,China |
| FNGS0311 | IV | Changchun, Jilin,China |
| FNGS0312 | I | Harbin, Heilongjiang, China |
| FNGS0313 | I | Harbin, Heilongjiang, China |
| FNGS0314 | I | Changchun, Jilin,China |
| FNGS0315 | I | Harbin, Heilongjiang, China |
| FNGS0316 | I | Harbin, Heilongjiang, China |
| FNGS0317 | I | Harbin, Heilongjiang, China |
| FNGS0318 | I | Harbin, Heilongjiang, China |
| FNGS0319 | I | Harbin, Heilongjiang, China |
| FNGS0320 | I | Harbin, Heilongjiang, China |
| FNGS0321 | II | Harbin, Heilongjiang, China |
| FNGS0322 | II | Harbin, Heilongjiang, China |
| FNGS0323 | II | Harbin, Heilongjiang, China |
| FNGS0324 | II | Harbin, Heilongjiang, China |
| FNGS0325 | II | Changchun, Jilin,China |
| FNGS0326 | V | Changchun, Jilin,China |
| FNGS0327 | V | Jilin,Jilin,China |
| FNGS0328 | V | Jilin,Jilin,China |
| FNGS0329 | V | Changchun, Jilin,China |
| FNGS0330 | III | Changchun, Jilin,China |
| FNGS0331 | V | Changchun, Jilin,China |
| FNGS0332 | III | Changchun, Jilin,China |
| FNGS0333 | V | Changchun, Jilin,China |
| FNGS0334 | III | Changchun, Jilin,China |
| FNGS0335 | V | Changchun, Jilin,China |
| FNGS0336 | V | Changchun, Jilin,China |
| FNGS0337 | III | Changchun, Jilin,China |
| FNGS0338 | V | Harbin, Heilongjiang, China |
| FNGS0339 | II | Suihua, Heilongjiang, China |
| FNGS0340 | IV | Changchun, Jilin,China |
| FNGS0341 | II | Changchun, Jilin,China |
| FNGS0342 | I | Changchun, Jilin,China |
| FNGS0343 | IV | Changchun, Jilin,China |
| FNGS0344 | V | Changchun, Jilin,China |
| FNGS0347 | II | Changchun, Jilin,China |
| FNGS0348 | II | Harbin, Heilongjiang, China |
| FNGS0349 | I | Jiamusi, Heilongjiang, China |
| FNGS0424 | II | Harbin, Heilongjiang,China |
| FNGS0425 | I | Jiamusi, Heilongjiang,China |
| FNGS0426 | I | Beian, Heilongjiang, China |
| FNGS0427 | II | Jiamusi, Heilongjiang,China |
| FNGS0428 | I | Heihe, Heilongjiang, China |
| FNGS0429 | V | Changchun, Jilin,China |
| FNGS0430 | II | Suihua, Heilongjiang, China |
| FNGS0431 | I | Changchun, Jilin,China |
| FNGS0853 | I | Beian, Heilongjiang, China |
| FNGS0854 | I | Beian, Heilongjiang, China |
| FNGS0855 | V | Changchun, Jilin,China |
| FNGS0856 | IV | Changchun, Jilin,China |

**Table S2:** List of soybean cultivars used for the qRT-PCR analysis with their branch number per plant in combined environment and classification.

| **Cultivar name** | **Number of branches per plant（combined environment）** | **Classification** |
| --- | --- | --- |
| FNGS0182 | 1.97 | High branch number |
| FNGS0201 | 2.13 | High branch number |
| FNGS0281 | 1.73 | High branch number |
| FNGS0853 | 2.07 | High branch number |
| FNGS0208 | 0.17 | Low branch number |
| FNGS0212 | 0.23 | Low branch number |
| FNGS0237 | 0.27 | Low branch number |
| FNGS0250 | 0.2 | Low branch number |
| FNGS0297 | 0.13 | Low branch number |
|  |  |  |

**Supplementary Table S3:** Primers used for the qRT-PCR analysis in this study.

| **Primer name** | **Primer sequence (5’- 3’)** | **Annotation** |
| --- | --- | --- |
| OL20293 F | TAGCCCAACAGGCACAACT | Glyma.04G159300 |
| OL20294 R | TAGGACGAAGCATCCAAGGT |  |
| OL20297 F | TCCTCCGTGGTGAATG | Glyma.12G073300 |
| OL20298 R | GACGTAGCGTGTCCTTTA |  |
| OL20301 F | AAGTTGGCTTGATAGTGGG | Glyma.12G074600 |
| OL20302 R | TTCCTGTTGTGGCATTAGA |  |
| OL20303 F | GCATGCACCATCGTTCTACG | Glyma.12G075300 |
| OL20304 R | CGCATGTCCCAGTTTTGCTT |  |
| OL20309 F | GGCTTGCGATTATCGTTG | Glyma.13G039600 |
| OL20310 R | GGCACTTGGGAGTTCCTCT |  |
| OL20321 F | TCGCTGCCATAAGTGAGTC | Glyma.20G060400 |
| OL20322 R | CTGGTCCAGAATACAAGGTTCG |  |
| OL20370 F  OL20371 R | GGTGGTTCTATCTTGGCATC  CTTTCGCTTCAATAACCCTA | GmActin11 q-PCR  (Glyma.18G290800) |

**Table S4:** Phenotypic analysis of branch number evaluated in 200 diverse soybean cultivars.

| **Environment** | **Minimum** | **Maximum** | **Mean±SE** | **SD** | **CV%** | **Skewness** | **Kurtosis** | ***h^2^*** |
| --- | --- | --- | --- | --- | --- | --- | --- | --- |
| E1 | 0.00 | 2.40 | 0.50 ±0.04 | 0.6 | 121.92 | 1.32 | 0.94 |  |
| E2 | 0.00 | 3.60 | 0.96±0.05 | 0.66 | 68.44 | 1.29 | 1.36 |  |
| E3 | 0.00 | 2.90 | 0.61±0.04 | 0.57 | 93.14 | 1.53 | 2.41 |  |
| CE | 0.00 | 3.60 | 0.69±0.03 | 0.64 | 92.75 | 1.28 | 1.50 | 0.61 |

**Table S5:** Distribution of all the SNPs used for GWAS across soybean chromosomes.

| Chromosome | Length(bp) | Length (Mb) | Numbers of SNPs | Inter-marker distance (bp) | Density (SNPs/Mb) |
| --- | --- | --- | --- | --- | --- |
| Chr01 | 56,831,624 | 56.83 | 126,955 | 447.65 | 2233.94 |
| Chr02 | 48,577,505 | 48.58 | 104,013 | 467.03 | 2141.07 |
| Chr03 | 45,779,781 | 45.78 | 149,020 | 307.21 | 3255.13 |
| Chr04 | 52,389,146 | 52.39 | 138,384 | 378.58 | 2641.42 |
| Chr05 | 42,234,498 | 42.23 | 68,939 | 612.64 | 1632.47 |
| Chr06 | 51,416,486 | 51.42 | 156,561 | 328.41 | 3044.75 |
| Chr07 | 44,630,646 | 44.63 | 120,352 | 370.83 | 2696.66 |
| Chr08 | 47,837,940 | 47.84 | 107,322 | 445.74 | 2243.35 |
| Chr09 | 50,189,764 | 50.19 | 142,206 | 352.94 | 2833.35 |
| Chr10 | 51,566,898 | 51.57 | 134,820 | 382.49 | 2614.31 |
| Chr11 | 34,766,867 | 34.77 | 53,502 | 649.82 | 1538.74 |
| Chr12 | 40,091,314 | 40.09 | 89,221 | 449.35 | 2225.52 |
| Chr13 | 45,874,162 | 45.87 | 131,517 | 348.81 | 2867.17 |
| Chr14 | 49,042,192 | 49.04 | 122,644 | 399.87 | 2500.90 |
| Chr15 | 51,756,343 | 51.76 | 211,191 | 245.07 | 4080.20 |
| Chr16 | 37,887,014 | 37.89 | 162,890 | 232.59 | 4299.02 |
| Chr17 | 41,641,366 | 41.64 | 151,558 | 274.76 | 3639.72 |
| Chr18 | 58,018,742 | 58.02 | 218,699 | 265.29 | 3769.37 |
| Chr19 | 50,746,916 | 50.75 | 205,967 | 246.38 | 4058.46 |
| Chr20 | 47,904,181 | 47.90 | 119,849 | 399.70 | 2502.07 |
| Total | 949,183,385 | 949.19 | 2,715,610 | 349.53 | 2860.98 |

**Table S6:** Significant *QTN* ×*environment* interactions (QEIs) of branch number in three environments (JMS17, JMS18 & JL20) detected using the QTN-by-environment detection model in 3VmrMLM.

| Marker | Chr | Position (bp) | LOD (QE) | add*JMS17 | dom*JMS17 | add*JMS18 | dom*JMS18 | add*JL20 | dom*JL20 | variance | r^2^(%) | P-value | significance |
| --- | --- | --- | --- | --- | --- | --- | --- | --- | --- | --- | --- | --- | --- |
| Chr18_32068331 | 18 | 32068331 | 6.1149 | 0.0496 | - | 0.1044 | - | -0.154 | - | 0.0124 | 3.0133 | 7.6793E-07 | SIG |

*“add” represent the additive effect, “dom” represent dominance effect.*

**Table S7**: Mean distribution of the haplotype allele groups underlying each haplotype block among the 200 soybean genotypes

| Hap | SNP1 | SNP2 | SNP3 | SNP4 |  |  | n | mean | significant difference |
| --- | --- | --- | --- | --- | --- | --- | --- | --- | --- |
| Hap4_1 | A/G | C/T | T/C | C/T |  |  | 173 | 0.41 | b |
| Hap4_2 | A/A | C/C | T/T | C/C |  |  | 22 | 1.15 | a |
| Hap4_3 | A/A | C/C | T/T | C/T |  |  | 5 | 0.60 | ab |
|  |  |  |  |  |  |  |  |  |  |
|  |  |  |  |  |  |  |  |  |  |
| Hap | SNP1 | SNP2 | SNP3 | SNP4 |  |  | n | mean | significant difference |
| Hap7_1 | T/C | C/A | A/G | T/C |  |  | 191 | 0.57 | b |
| Hap7_2 | T/T | C/A | A/G | T/C |  |  | 6 | 0.57 | b |
| Hap7_3 | T/T | C/C | A/A | T/T |  |  | 3 | 2.30 | a |
|  |  |  |  |  |  |  |  |  |  |
|  |  |  |  |  |  |  |  |  |  |
| Hap | SNP1 | SNP2 | SNP3 | SNP4 |  |  | n | mean | significant difference |
| Hap12_1 | C/A | A/G | A/G | C/T |  |  | 159 | 0.46 | b |
| Hap12_2 | C/C | A/A | A/G | C/T |  |  | 22 | 0.56 | b |
| Hap12_3 | C/A | A/A | A/G | C/T |  |  | 15 | 0.41 | b |
| Hap12_4 | C/A | A/G | A/A | C/C |  |  | 4 | 1.85 | a |
|  |  |  |  |  |  |  |  |  |  |
|  |  |  |  |  |  |  |  |  |  |
| Hap | SNP1 | SNP2 | SNP3 | SNP4 | SNP5 |  | n | mean | significant difference |
| Hap13A_1 | G/A | C/C | G/G | G/G | G/A |  | 64 | 0.70 | a |
| Hap13A_2 | G/G | T/T | A/A | G/A | G/G |  | 67 | 0.30 | b |
| Hap13A_3 | G/A | C/C | A/G | G/G | G/A |  | 31 | 0.25 | b |
| Hap13A_4 | A/A | C/C | G/G | G/G | G/A |  | 20 | 0.61 | ab |
| Hap13A_5 | A/A | C/C | A/G | G/G | G/A |  | 18 | 0.68 | ab |
|  |  |  |  |  |  |  |  |  |  |
|  |  |  |  |  |  |  |  |  |  |
| Hap | SNP1 | SNP2 | SNP3 | SNP4 |  |  | n | mean | significant difference |
| Hap13B_1 | G/A | A/T | G/A | A/G |  |  | 189 | 0.46 | b |
| Hap13B_2 | G/A | A/T | G/G | A/A |  |  | 7 | 0.54 | b |
| Hap13B_3 | G/G | A/A | G/G | A/A |  |  | 4 | 2.05 | a |
|  |  |  |  |  |  |  |  |  |  |
|  |  |  |  |  |  |  |  |  |  |
| Hap | SNP1 | SNP2 |  |  |  |  | n | mean | significant difference |
| Hap16_1 | A/A | A/A |  |  |  |  | 187 | 0.55 | b |
| Hap16_2 | G/G | G/G |  |  |  |  | 13 | 1.35 | a |
|  |  |  |  |  |  |  |  |  |  |
|  |  |  |  |  |  |  |  |  |  |
| Hap | SNP1 | SNP2 | SNP3 | SNP4 | SNP5 | SNP6 | n | mean | significant difference |
| Hap17_1 | A/G | A/G | A/G | T/G | G/A | G/A | 191 | 0.67 | b |
| Hap17_2 | A/G | A/G | A/A | T/T | G/G | G/G | 5 | 0.68 | b |
| Hap17_3 | A/A | A/A | A/A | T/T | G/G | G/G | 4 | 1.62 | a |
|  |  |  |  |  |  |  |  |  |  |
|  |  |  |  |  |  |  |  |  |  |
| Hap | SNP1 | SNP2 | SNP3 | SNP4 | SNP5 |  | n | mean | significant difference |
| Hap18_1 | A/G | T/G | A/T | G/A | C/T |  | 147 | 0.84 | b |
| Hap18_2 | A/A | T/G | A/T | G/A | C/T |  | 21 | 0.92 | b |
| Hap18_3 | A/A | T/T | A/A | G/G | C/C |  | 27 | 1.7 | a |
| Hap18_4 | A/A | T/T | A/A | G/A | C/T |  | 5 | 0.88 | b |
|  |  |  |  |  |  |  |  |  |  |
|  |  |  |  |  |  |  |  |  |  |
| Hap | SNP1 | SNP2 | SNP3 |  |  |  | n | mean | significant difference |
| Hap19A_1 | G/G | T/T | A/A |  |  |  | 133 | 0.33 | b |
| Hap19A_2 | G/G | C/C | G/G |  |  |  | 26 | 0.75 | a |
| Hap19A_3 | A/A | C/C | G/G |  |  |  | 23 | 0.96 | a |
| Hap19A_4 | G/G | C/T | G/A |  |  |  | 18 | 0.72 | a |
|  |  |  |  |  |  |  |  |  |  |
|  |  |  |  |  |  |  |  |  |  |
| Hap | SNP1 | SNP2 | SNP3 | SNP4 | SNP5 | SNP6 | n | mean | significant difference |
| Hap19B_1 | G/G | C/C | T/T | C/C | T/T | G/G | 164 | 0.64 | b |
| Hap19B_2 | G/T | C/T | T/A | C/G | T/A | G/A | 36 | 0.94 | a |
|  |  |  |  |  |  |  |  |  |  |
|  |  |  |  |  |  |  |  |  |  |
| Hap | SNP1 | SNP2 |  |  |  |  | n | mean | significant difference |
| Hap20_1 | C/T | C/T |  |  |  |  | 90 | 0.43 | b |
| Hap20_2 | C/T | C/C |  |  |  |  | 81 | 0.40 | b |
| Hap20_3 | C/C | C/C |  |  |  |  | 10 | 1.34 | a |
| Hap20_4 | C/C | T/T |  |  |  |  | 12 | 1.13 | a |
| Hap20_5 | C/T | T/T |  |  |  |  | 7 | 0.17 | b |

**Table S8:** Variant annotation analysis of the eleven significant SNPs.

| Significant SNP | Chromosome | SNP position | Reference/Altered | SNP annotation |
| --- | --- | --- | --- | --- |
| Chr04_39125623 | Chr04 | 39125623 | A/G | Intergenic region |
| Chr07_29399935 | Chr07 | 29399935 | A/G | Intergenic region |
| Chr12_5601871 | Chr12 | 5601871 | C/T | Synonymous variant |
| Chr13_12082492 | Chr13 | 12082492 | A/G | Intergenic region |
| Chr13_17432336 | Chr13 | 17432336 | A/T | Nonsynonymous variant |
| Chr16_6096184 | Chr16 | 6096184 | A/G | Intergenic region |
| Chr17_38943942 | Chr17 | 38943942 | T/G | Intergenic region |
| Chr18_32068331 | Chr18 | 32068331 | C/T | Intergenic region |
| Chr19_10711770 | Chr19 | 10711770 | G/A | Intergenic region |
| Chr19_11321785 | Chr19 | 11321785 | T/A | Intergenic region |
| Chr20_18794801 | Chr20 | 18794801 | C/T | Intergenic region |
